# Exceptional Enhancement of Optical Anisotropy Achieved via the Strategy of Combining Rigid Groups with High Symmetry and π‐Conjugated Organic Groups in Hybrid Fluorides

**DOI:** 10.1002/advs.202515170

**Published:** 2025-10-06

**Authors:** Ru‐Ling Tang, Bing‐Wei Miao, Guo‐Ren Zhu, Wenlong Liu, Sheng‐Ping Guo

**Affiliations:** ^1^ School of Chemistry and Chemical Engineering Yangzhou University Yangzhou 225002 P. R. China; ^2^ School of Materials and Energy Yunnan Key Laboratory of Electromagnetic Materials and Devices Yunnan University Kunming 650500 P. R. China

**Keywords:** birefringence, fluorosilicates, organic and inorganic hybrid halides, rigid group

## Abstract

Birefringent crystal materials used for modulating light polarization are of great significance in optical communication applications. According to the numerous reported organic–inorganic hybrid optical crystal materials, adopting π‐conjugated groups with high polarizability anisotropy is deemed to an effective strategy for constructing excellent birefringent crystals, while how to make the organic groups align in parallel to produce superimposed optical anisotropy remains a challenge. Here, an effective strategy of combining the inorganic rigid spherical group MF_6_ (M = Si, Ge, Ga, Al) is implemented with the organic π‐conjugated group to promote coplanar alignment, and obtained two organic–inorganic hybrid fluorosilicate birefringent crystals, (C_6_H_5_N_2_)_2_SiF_6_ and (C_10_H_10_N_2_)SiF_6_. It's worth noting that their crystal structures are constructed by organic cations and [SiF_6_]^2−^ anions with N‐H···F hydrogen bonds as bridges. The organic cations in both compounds are arranged in parallel in multiple directions under the guidance of the rigid group SiF_6_ units. This optimal arrangement enables them to exhibit excellent optical anisotropy. (C_10_H_10_N_2_)SiF_6_ shows the largest experimental birefringence (Δ*n*) of 0.583@546 nm among known fluorides containing rigid groups. (C_6_H_5_N_2_)_2_SiF_6_ also exhibits a superior birefringence (Δ*n*_exp._ = 0.505@546 nm), far outperforming all current commercial birefringent crystals, which make them excellent candidates for birefringent crystal materials.

## Graphical abstract

We successfully synthesized two types of strong optically anisotropic organic‐inorganic hybrid fluorosilicate birefringent crystals: (C_6_H_5_N_2_)_2_SiF_6_ and (C_10_H_10_N_2_)SiF_6_, by adopting a strategy where the inorganic rigid groups SiF_6_ guided the parallel arrangement of the organic π‐conjugated groups. Their experimental birefringences at 546 nm are 0.583 and 0.505, respectively, both of which are far superior to the existing commercial birefringent crystals.

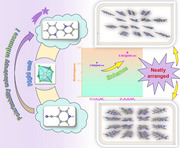

## 1 Introduction

Birefringent crystal materials, characterized by their unique optical anisotropy, have the remarkable ability to split incident light into two refracted beams with distinct propagation velocities and polarization states. This inherent property renders them indispensable fundamental components in modern optics and optoelectronics. From conventional waveplates and polarization beam splitters to cutting‐edge integrated optical devices and quantum communication systems, birefringent crystals play a pivotal role in critical technologies such as beam manipulation, optical signal processing, and quantum state control.^[^ ^1^, ^2^, ^3^ ^]^ Nevertheless, with the rapid evolution of photonics toward higher integration and superior performance, the existing limitations of commercial birefringent crystals (MgF_2_ (0.012@546 nm), *α*‐BaB_2_O_4_ (0.1238@546 nm),^[^ ^4^ ^]^ CaCO_3_ (0.172@564 nm), YVO_4_ (0.204@532 nm),^[^ ^5^ ^]^ and LiNbO_3_ (0.074@546 nm)^[^ ^6^ ^]^ encounter several critical challenges. Natural crystals often suffer from compromised quality, as defects and impurities disrupt the optical homogeneity, leading to inconsistent polarization manipulation. In the case of 𝛼‐BaB_2_O_4_, its pronounced anisotropic thermal expansion can cause dimensional instability under temperature variations, which is detrimental to precision optical systems. YVO_4_ exhibits suboptimal ultraviolet (UV) transmittance, severely restricting its application in UV‐related optical devices. Moreover, LiNbO_3_ and MgF_2_’s relatively low birefringences fail to meet the stringent requirements of high‐performance polarization‐based technologies.^[^ ^7^, ^8^ ^]^ Consequently, breakthroughs in material innovation and fabrication techniques are urgently required to achieve substantial performance enhancements.^[^ ^9^, ^10^, ^11^ ^]^

In recent years, several effective strategies have emerged for the design and synthesis of birefringent crystal materials. The prominent approach involves rational crystal structure design, where researchers focus on incorporating anisotropic building blocks. The most effective route is to introduce strongly polarizable building units. One potent strategy for achieving substantial optical anisotropy entails integrating π‐conjugated planar groups or distorted metal cationic coordination polyhedra.^[^ ^12^, ^13^, ^14^, ^15^ ^]^ Initially, researchers mainly focused on inorganic planar units, such as [BO_3_]^3^^−^, [CO_3_]^2^−, as well as cations including Sn^2+^, Pb^2+^, Sb^3+^, V^5+^, Nb^5+^ and Mo^6+^ into molecular frameworks.^[^ ^16^, ^17^, ^18^ ^]^ Representative examples of crystals developed via this approach include *α*‐BaB_2_O_4_,^[^ ^4^ ^]^ Ca(BO_2_)_2_,^[^ ^8^ ^]^ RbSn_2_Cl_5_,^[^ ^19^ ^]^ Al_2_Te_2_MoO_10_,^[^ ^20^ ^]^ MgTeMoO_6_,^[^ ^21^ ^]^ K_2_SbMoO_2_F_7_,^[^ ^22^ ^]^ and RbSbF_2_SeO_4_.^[^ ^23^ ^]^ Although these structural units effectively induce birefringence, the magnitude of birefringence in the resulting materials typically remains below 0.3, indicating room for further enhancement in optical performance. Subsequently, considering the similarity of many π‐conjugated organic planar groups with inorganic planar units, many high‐performance organic‐inorganic hybrid birefringent materials emerged,^[^ ^24^ ^]^ such as Li_2_(HC_3_N_3_S_3_)·5H_2_O (Δ*n*_exp._ = 0.532@546 nm),^[^ ^25^ ^]^ C_9_H_7_NBrNO_3_ (Δ*n*_exp._ = 0.401@550 nm),^[^ ^26^ ^]^ (C6H6NO2)^+^Cl^−^ (Δ*n*_exp._ = 0.363@550 nm),^[^ ^5^ ^]^ {N[C(NH_2_)_2_]_2_}_2_CO_3_ (Δ*n*_exp._ = 0.232@550 nm),^[^ ^27^ ^]^ (C_2_N_3_H_4_)_2_PbCl_4_ (Δ*n*_exp._ = 0.18@550 nm),^[^ ^16^ ^]^ and CN_4_H_7_SO_3_CF_3_ (Δ*n*_cal._ = 0.149@546 nm).^[^ ^17^ ^]^ However, the π‐conjugated organic groups may not always play a very positive role. Hence, an advanced structural design strategy has yet to be conceived for the synthesis of high‐performance birefringent crystals.

**Figure 1:**
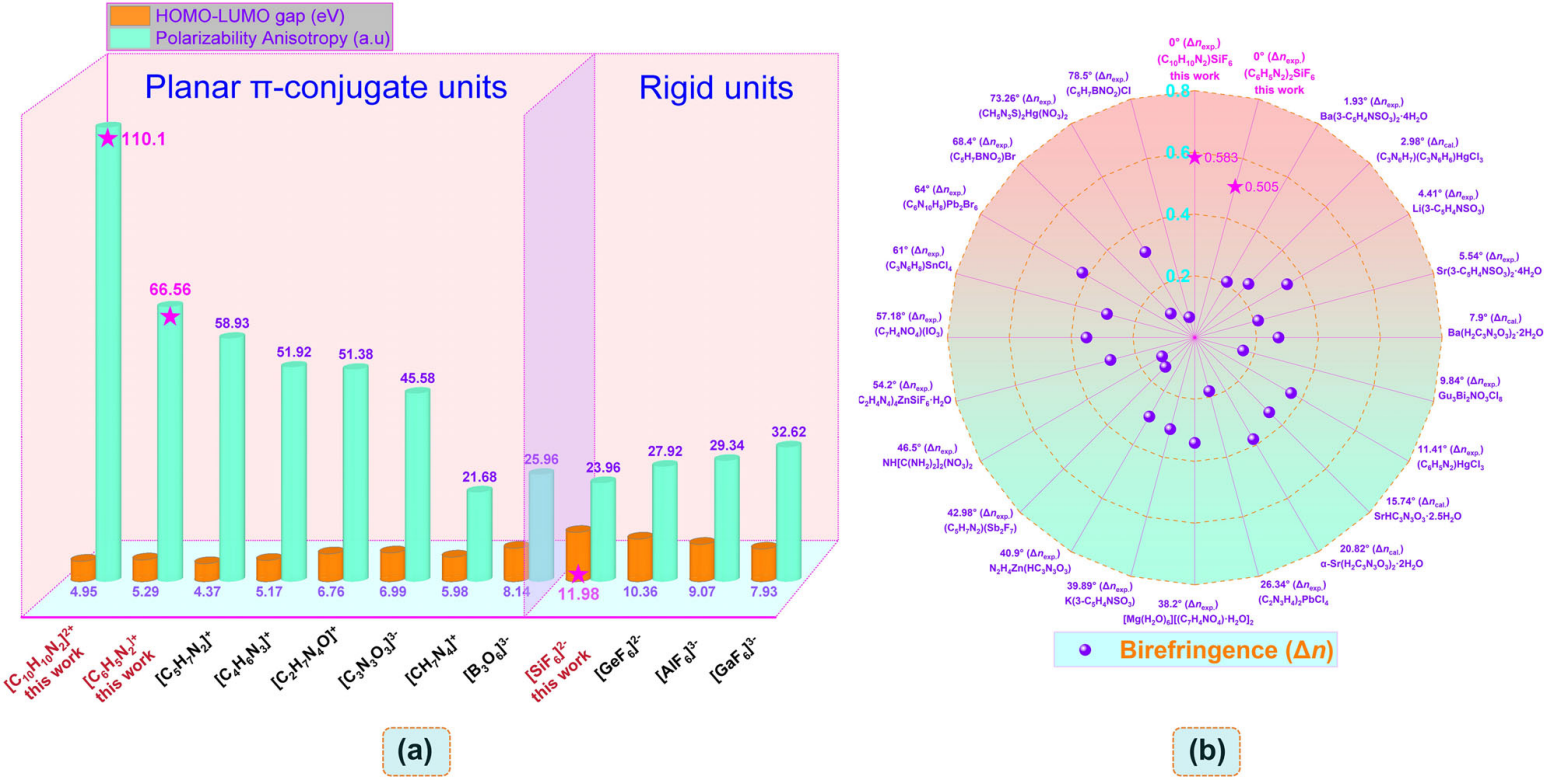
a) Polarizability anisotropy (δ) and HOMO‐LUMO gaps of representative π‐conjugated groups and rigid units. b) Birefringence of organic‐inorganic hybrid materials and dihedral angles between organic π‐conjugated units in crystal structures.

It is well known that, in addition to the strong optical anisotropy inherent in the structural unit, the cornerstone of attaining substantial birefringence resides in the high packing density and the ordered arrangement of structural units. Several factors cause to the challenging ordered arrangement of anisotropic structural units. First, kinetic limitations in crystal growth processes often impede the precise positioning of these units. Second, thermodynamic instability can play a crucial role. Anisotropic units with high surface energy or complex coordination requirements may preferentially form metastable structures rather than the desired ordered arrangements.^[^ ^17^ ^]^ Third, intermolecular or interatomic interactions between anisotropic units can be highly complex. The presence of multiple types of bonding forces, such as hydrogen bonds, van der Waals forces, and electrostatic interactions, may compete with each other, disrupting the regular assembly of the units.^[^ ^18^, ^28^, ^29^, ^30^, ^31^ ^]^ Especially in the organic‐inorganic hybrid material system, the inorganic units with low symmetry are difficult to promote the complete parallel arrangement of the organic planar units, which is very common in the recently reported birefringent crystal materials, such as (C_6_H_5_N_2_)HgCl_3_,^[^ ^32^ ^]^ Li_2_(HC_3_N_3_S_3_)·5H_2_O,^[^ ^25^ ^]^ (C_12_H_8_N_2_)SbF_2_(H_2_PO_3_),^[^ ^33^ ^]^ (C_3_H_5_N_2_)SbF_2_SO_4_,^[^ ^34^ ^]^ and (C_6_N_10_H_8_)Pb_2_Br_6_.^[^ ^35^ ^]^ In addition, the magnitude of the dihedral angle exerts a profound influence on optical anisotropy. A smaller dihedral angle typically leads to a more planar molecular conformation, facilitating effective π‐electron delocalization across adjacent structural units. This enhanced conjugation significantly modifies the electron cloud distribution, thereby increasing the refractive index differences along different crystallographic axes and boosting optical anisotropy. Additionally, the dihedral angle affects intermolecular packing arrangements; optimized dihedral angles can promote the formation of highly ordered crystal structures, where the alignment of anisotropic molecules maximizes optical property differences. We have summed up some recently reported organic‐inorganic hybrid birefringent materials and provided the dihedral angles between the organic π‐conjugated units in their crystal structures (**Figure** **1**b).^[^ ^36^, ^37^, ^38^, ^39^, ^40^, ^41^, ^42^, ^43^, ^44^ ^]^ It can be found that a smaller dihedral angle, especially close to 0° (when the organic π‐conjugated units are arranged in a completely parallel pattern),^[^ ^29^, ^31^ ^]^ is more conducive to improving birefringence.^[^ ^45^ ^]^ Based on the above considerations, we have decided to adopt spherically symmetric octahedral rigid units, such as SiF_6_ and AlF_6_, which can effectively promote the formation of a higher degree of ordered arrangement of organic groups to maximize the differences in optical properties and maintain a wide band gap with the highly electronegative F atoms. This phenomenon can be attributed to several underlying mechanisms. First, the geometric symmetry of these octahedral units creates a uniform spatial environment around them. The equidistant arrangement of six ligands in an octahedron generates a consistent steric hindrance pattern. As organic groups interact with these octahedral units, the steric repulsion forces exerted by the ligands guide the organic molecules to adopt a parallel orientation to minimize spatial conflicts, ensuring an energetically favorable packing arrangement.^[^ ^20^, ^46^ ^]^ Second, the electrostatic interaction between the octahedral units and organic groups plays a crucial role. The charged nature of the octahedral complexes, often with a defined charge distribution, can establish specific electrostatic attractions or repulsions with functional groups on the organic molecules. These electrostatic forces encourage the alignment of organic groups in a parallel fashion.^[^ ^17^ ^]^ Moreover, the rigidity of the octahedral structure prevents significant deformation under external forces or during crystal growth, maintaining a stable framework that supports the parallel alignment of organic groups and contributes to the formation of highly ordered molecular assemblies with enhanced optical and physical properties.^[^ ^47^, ^48^, ^49^, ^50^ ^]^

Hence, under the strategy of combining rigid fluorination units and π‐conjugated organic groups,^[^ ^51^ ^]^ we first obtained a new hybrid fluoride (C_6_H_5_N_2_)_2_SiF_6_ in aqueous solution. The π‐conjugated [C_6_H_5_N_2_]^+^ cations present the optimal completely parallel arrangement under the guidance of the rigid groups. Given that the combination of an organic π‐conjugated unit with six‐membered ring and an inorganic rigid group exhibits such excellent linear optical properties, we have further conducted experiments using an organic ligand (2‐2′‐bipyridine) with a double six‐membered ring and higher polarizability anisotropy instead of 4‐cyanopyridine, and obtained another hybrid fluoride (C_10_H_10_N_2_)SiF_6_. In this work, the crystal structures, optical properties, especially optical anisotropy and structural‐performance analyses based on crystal structures and theoretical calculations of these two compounds are presented. Our work not only introduces two new high‐performance birefringent materials, but also provides new ideas for exploring high‐performance fluoride birefringent crystal materials.

## 2 Results and Discussion

**Figure 2:**
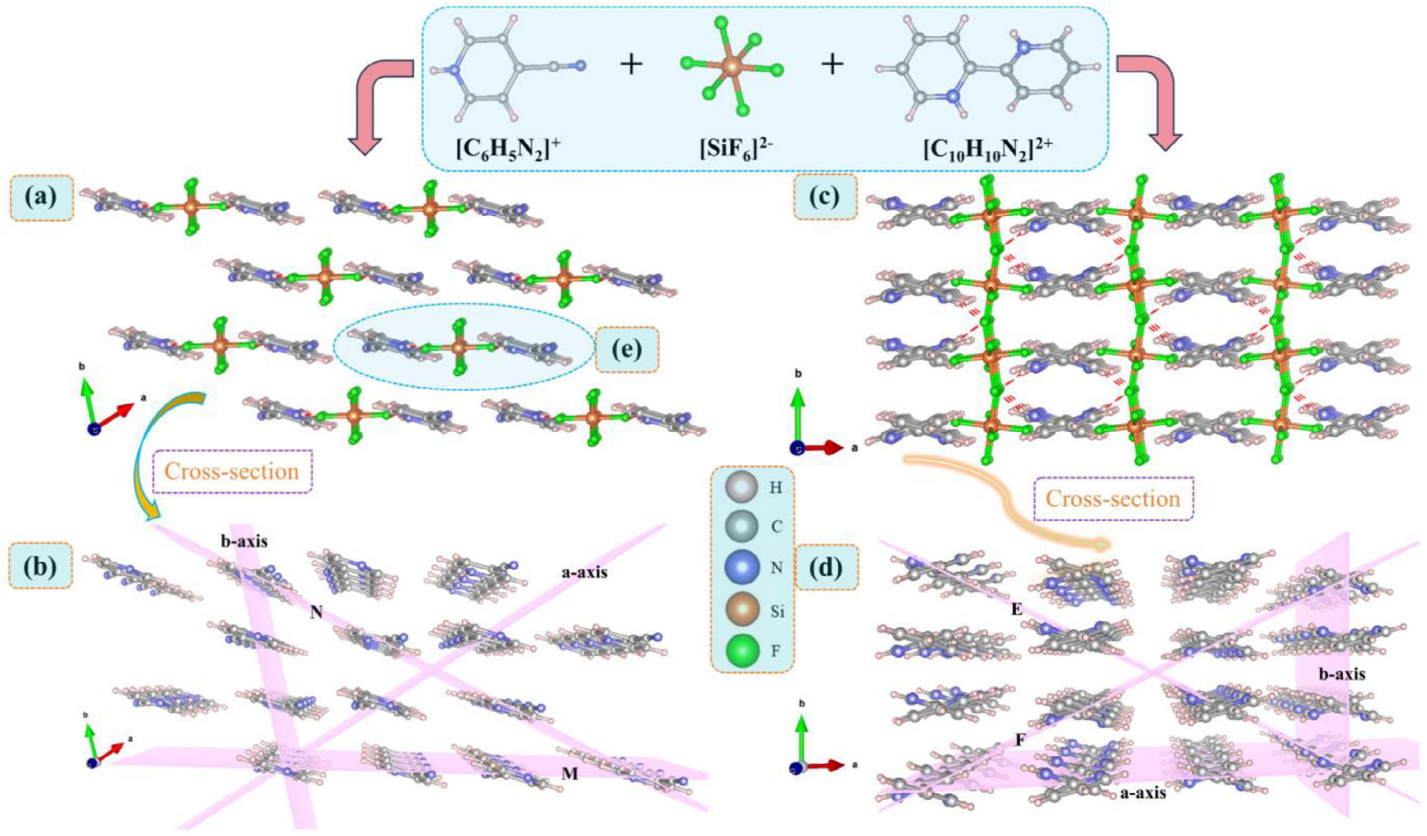
a) The crystal structure of (C_6_H_5_N_2_)_2_SiF_6_ observed along the c‐axis and rotated counterclockwise by 33.3°. b) The [C_6_H_5_N_2_]^+^ cations are arranged in parallel along the M, N and coordinate axes, respectively. c) The crystal structure of (C_10_H_10_N_2_)SiF_6_ along the c‐axis. d) The [C_10_H_10_N_2_]^2+^ cations are arranged in parallel along the E, F and coordinate axes respectively. e) The repeating unit of (C_6_H_5_N_2_)_2_SiF_6_.

It is known that planar π‐conjugated groups usually have large polarizability anisotropy, which is conducive to large birefringence.^[^ ^17^ ^]^ This work employs aqueous solution evaporation technology to combine highly coordinated rigid inorganic SiF_6_ units with π‐conjugated 4‐cyanopyridine or 2,2′‐bipyridine organic ligands, respectively. As results, two hybrid fluorosilicates, (C_6_H_5_N_2_)_2_SiF_6_ and (C_10_H_10_N_2_)SiF_6_, with excellent birefringent properties were obtained. The colorless rod‐like crystals of (C_6_H_5_N_2_)_2_SiF_6_ and the colorless blocky crystals of (C_10_H_10_N_2_)SiF_6_ are shown in **Figure** **2**h,i. Their crystal structures were confirmed by single‐crystal X‐ray diffraction (XRD), with detailed crystallographic data provided in Tables S1–S3 (Supporting Information). Their phase purities were verified by powder XRD analysis (Figure S1, Supporting Information), confirming that their samples are suitable for further characterization and testing. Thermogravimetric analysis indicates that (C_6_H_5_N_2_)_2_SiF_6_ and (C_10_H_10_N_2_)SiF_6_ have good thermal stability, with the decomposition temperatures higher than 80 °C (Figure S5, Supporting Information).^[^ ^33^ ^]^ Energy‐dispersive X‐ray spectroscopy (EDS) tests were conducted on several crystals of them using Bruker quantum dispersive spectrometer. The EDS test results (Figure S4, Supporting Information) show that the elements C, N, Si and F are present in their crystals, and their proportions are close to the results of crystal structure determination.

**Figure 3:**
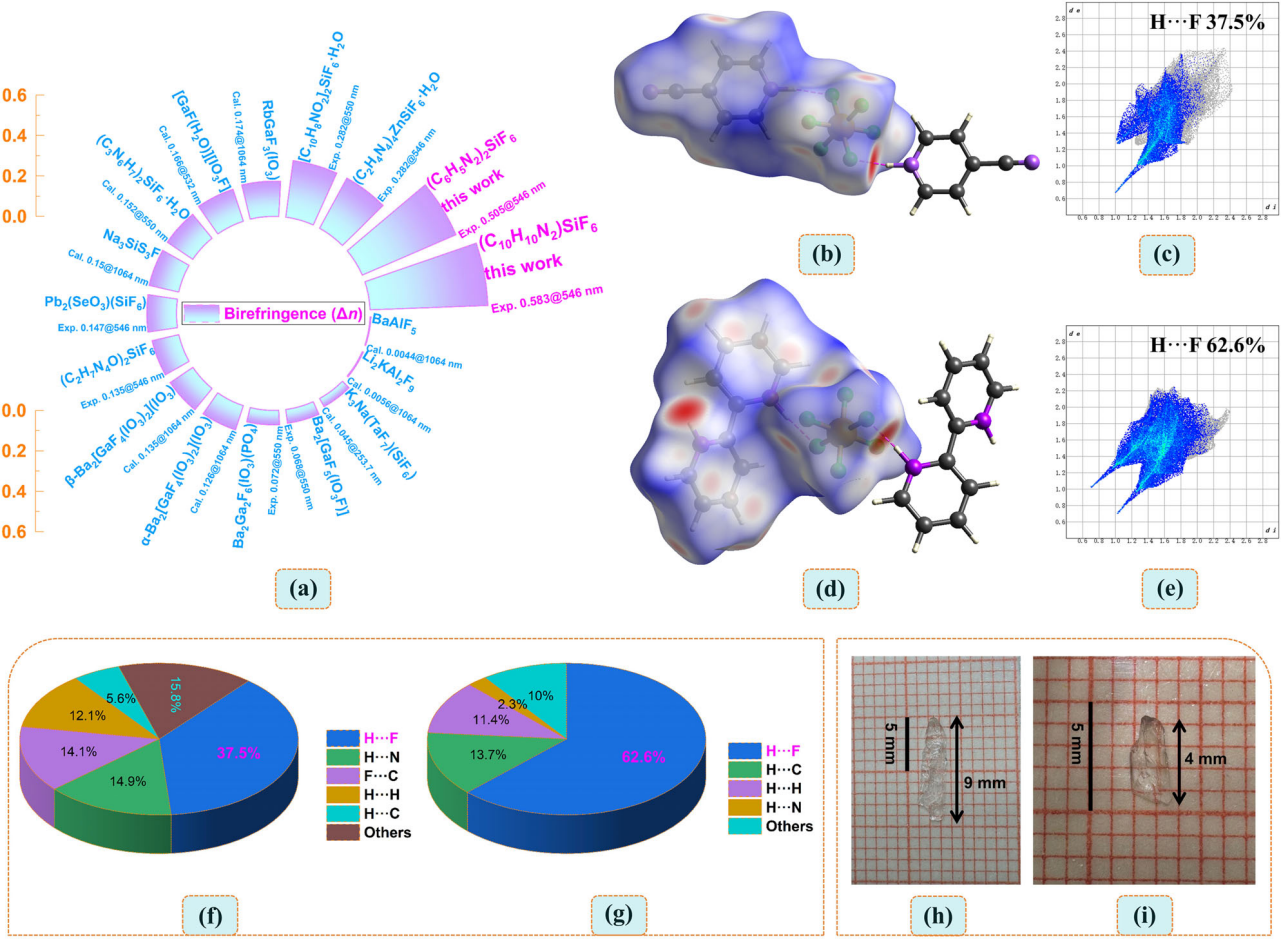
a) Birefringence comparison of reported crystal materials containing inorganic rigid groups. The hirshfeld surface analysis with *d*_norm_ for b) (C_6_H_5_N_2_)_2_SiF_6_ and d) (C_10_H_10_N_2_)SiF_6_. 2D fingerprint plots for individual interactions of hydrogen bond types in crystals of c) (C_6_H_5_N_2_)_2_SiF_6_ and e) (C_10_H_10_N_2_)SiF_6_. The relative contributions of various intermolecular contacts in f) (C_6_H_5_N_2_)_2_SiF_6_ and g) (C_10_H_10_N_2_)SiF_6_ crystals to the Hirschfeld surface areas. Crystals’ pictures of h) (C_6_H_5_N_2_)_2_SiF_6_, i) (C_10_H_10_N_2_)SiF_6_. Intermolecular contacts that are longer or shorter than the sum of van der Waals radii are depicted in blue and red on the *d*_norm_ surface, respectively. *d*_norm_ is the normalized contact distance. *d*_i_ and *d*_e_ are the distances from the Hirshfeld surface to the nearest nucleus inside and outside, respectively.

The crystal structure of (C_6_H_5_N_2_)_2_SiF_6_ was determined by single‐crystal XRD technique. It crystallizes in the centrosymmetric space group *P* $\bar{1}$ (No. 2) of the triclinic crystal system. The unit cell parameters of (C_6_H_5_N_2_)_2_SiF_6_ are as follows: a = 7.2153(6) Å, b = 7.3952(6) Å, c = 7.8907(7) Å, α = 79.296(5)°, β = 63.583(4)°, γ = 66.275(4)°, Z = 1, and V = 345.19(5) Å^3^. (C_6_H_5_N_2_)_2_SiF_6_ has a 0D structure, and its asymmetric unit includes a protonated 4‐cyanopyridine cation, a special Si atom and three unique F atoms. In the crystal structure of (C_6_H_5_N_2_)_2_SiF_6_, the two F atoms in each [SiF_6_]^2^^−^ group are connected to two protonated 4‐cyanopyridine cations through N‐H···F hydrogen bonds, forming a basic repeating unit (**Figure** **3**e), which are spread out parallel along the coordinate axes. The bond lengths of N‐H···F hydrogen bonds in (C_6_H_5_N_2_)_2_SiF_6_ is 1.823 Å (Figure S2, Supporting Information). Observing along the c‐axis, it can be found that a pseudo‐planar layer is formed in the direction of 33.3° clockwise rotation along the a‐axis (Figure 3a). Since the speed of light varies greatly in the two directions perpendicular to and parallel to the layered structure,^[^ ^32^ ^]^ it is conducive to excellent optical anisotropy.

**Figure 4:**
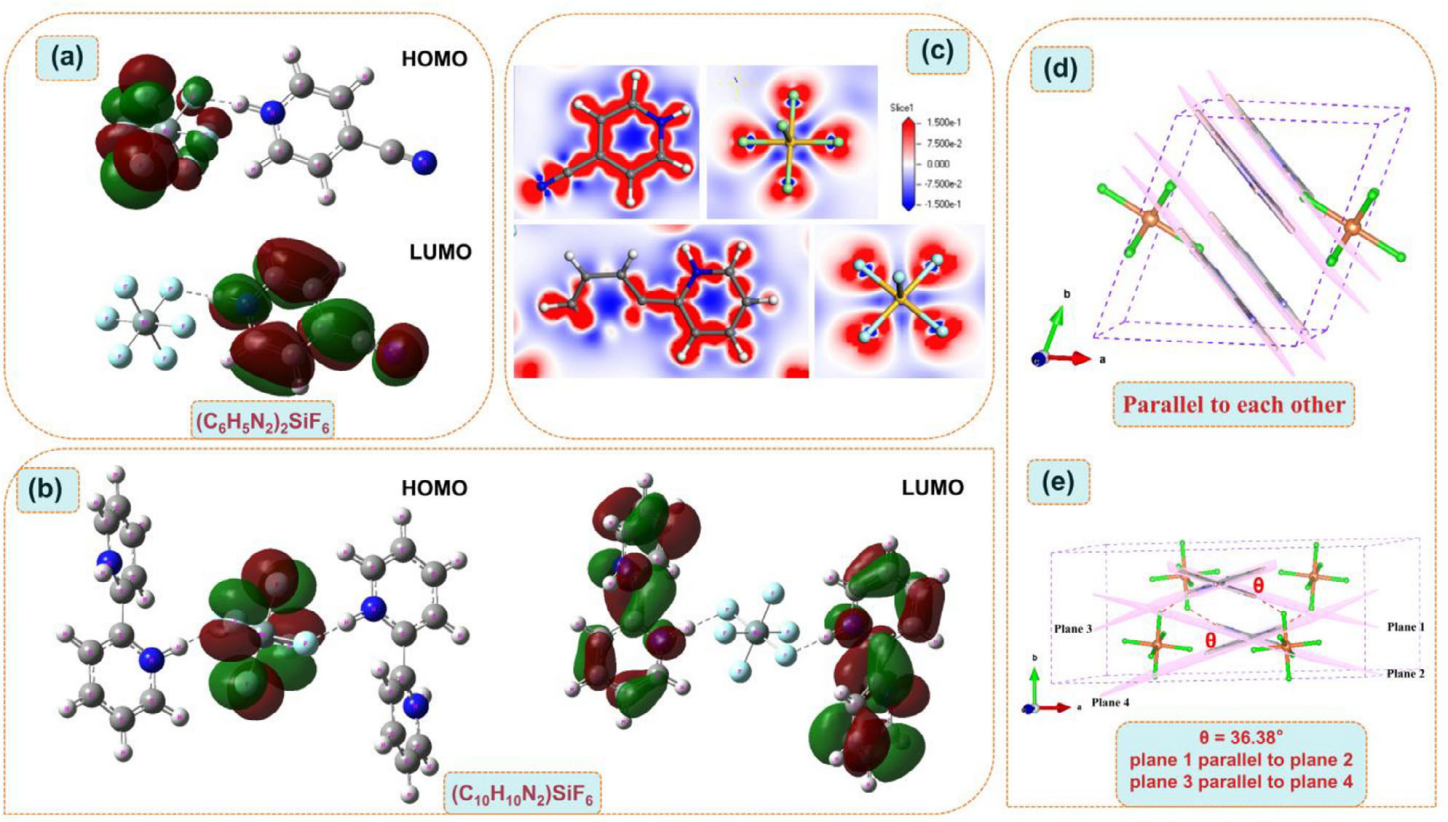
The local HOMO‐LUMO patterns of a) (C_6_H_5_N_2_)_2_SiF_6_ and b) (C_10_H_10_N_2_)SiF_6._ c) Differential charge density diagrams of [C_6_H_5_N_2_]^+^ cation, [C_10_H_10_N_2_]^2+^ cation and [SiF_6_]^2^^−^ anion in (C_6_H_5_N_2_)_2_SiF_6_ and (C_10_H_10_N_2_)SiF_6_. d) Distribution of the protonated planar [C_6_H_5_N_2_]^+^ units in (C_6_H_5_N_2_)_2_SiF_6_. e) Distribution of the protonated [C_10_H_10_N_2_]^2+^ units in (C_10_H_10_N_2_)SiF_6_ and the dihedral angle between the two six‐membered rings in the protonated [C_10_H_10_N_2_]^2+^ cation.

The crystal structure of (C_10_H_10_N_2_)SiF_6_ was also determined by single‐crystal XRD technique. It crystallizes in the centrosymmetric space group *C*2/*c* (No. 15) of the monoclinic crystal system. The unit cell parameters of (C_10_H_10_N_2_)SiF_6_ are as follows: a = 17.9015(19) Å, b = 6.6001(7) Å, c = 12.386(3) Å, α = γ = 90°, β = 131.249(2)°, Z = 4, and V = 1100.3(3) Å^3^. And the crystal structure of (C_10_H_10_N_2_)SiF_6_ has been previously reported by Marina S. Fonari et al.^[^ ^52^ ^]^ (C_10_H_10_N_2_)SiF_6_ has a 3D structure, and its asymmetric unit includes a protonated 2,2′‐bipyridine cation, a unique Si atom and three types of F atoms. The two six‐membered rings in the protonated 2,2′‐bipyridine cation are non‐coplanar, and the dihedral angle is 36.38° (**Figure** **4**e). Similarly, in (C_10_H_10_N_2_)SiF_6_, both N atoms in the π‐conjugated 2,2′‐bipyridine molecule have undergone protonation, providing more possibilities for the formation of hydrogen bonds. Structural analysis can reveal that in each protonated 2,2′‐bipyridine cationic group, the H(1) atoms connected to the two N(1) atoms all form N‐H···F hydrogen bonds with the [SiF_6_]^2^ ^−^ anionic group, thereby presenting a 3D structure (Figure 3c). The bond lengths of N‐H···F hydrogen bonds in (C_10_H_10_N_2_)SiF_6_ is 1.87 Å. The two N atoms in each 2,2′‐bipyridinecations present the trans nitrogen atom conformation. The distances between the C(4) and N(1) atoms on both sides of the [C_10_H_10_N_2_]^2+^ cation are 2.95 Å (Figure S2, Supporting Information). The same distance between the atoms on both sides indicates that it has a trans structure and is reasonable, which is consistent with the recent description of organic‐inorganic hybrid materials containing 2,2′‐bipyridine molecules, such as [bpy^2+^]_3_Pb_3_Br_10_·2Br.^[^ ^53^ ^]^

Overall, (C_6_H_5_N_2_)_2_SiF_6_ crystallizes in the triclinic crystal system with the lowest symmetry, corresponding to the space group *P* $\bar{1}$ (No. 2) and presenting a 0D structure. The lengths of its a, b, and c axes are approximately (7.215‐7.891 Å), presenting an overall compact feature of nearly cubic size. Moreover, the single‐ring 4‐cyanopyridine has a smaller molecular volume compared to the double‐ring 2,2′‐bipyridine, that is, (C_6_H_5_N_2_)_2_SiF_6_ behaves as a typical small molecule crystal. The unit density analyses of these two compounds shows that the density of the rigid [SiF_6_]^2^^−^ anion groups in both is close, while in (C_6_H_5_N_2_)_2_SiF_6_, two monoprotonated [C_6_H_5_N_2_]^+^ cations are required to balance the charge with it. Therefore, the result shows that the organic unit density in (C_6_H_5_N_2_)_2_SiF_6_ is larger (Table S8, Supporting Information). This also confirms the compact feature of similar axis lengths mentioned above. The compound (C_10_H_10_N_2_)SiF_6_ obtained through the 2,2′‐bipyridine substitution experiment crystallizes in the *C*2/*c* (No. 15) space group with higher symmetry in the monoclinic crystal system. Its typical characteristics are a longer a‐axis (17.9015(19) Å) and a larger β‐angle (131.249(2)°), which also indicates that it has a distinct growth trend along the a‐axis. Due to the larger volume of the 2,2′‐bipyridine molecule with a double ring and the fact that the diprotonated [C_10_H_10_N_2_]^2+^ cation provides more potential positions for hydrogen bond formation, the connection mode between the π‐conjugated organic group and the SiF_6_ unit is more diverse compared to (C_6_H_5_N_2_)_2_SiF_6_. That is, the crystal structure of (C_10_H_10_N_2_)SiF_6_ evolves from a 0D structure to a 3D structure, and it has a larger unit cell volume (V = 1100.3(3) Å^3^), and its Z value also increases from 1 to 4. Although the volume occupied by each chemical formula unit in (C_10_H_10_N_2_)SiF_6_ is less than that in (C_6_H_5_N_2_)_2_SiF_6_ (V/Z, 275.1 < 345.19 (Å^3^)), this indicates that the average contribution of each [C_10_H_10_N_2_]^2+^ cation in (C_10_H_10_N_2_)SiF_6_ to the volume is smaller. Structural analysis shows that the crystal structures of both compounds are based on rigid inorganic SiF_6_ units as bridging intermediates. The two F atoms in the [SiF_6_]^2^^−^ anionic group are respectively connected to the protonated 4‐cyanopyridine cation or 2,2′‐bipyridine cation by forming N‐H···F hydrogen bonds (Figure S2, Supporting Information), while maintaining the charge balance of the overall structure. This connection method maximizes the synergistic effect between rigid inorganic groups and organic components and plays a positive role in improving their linear optical performance. Interestingly, the planar π‐conjugated cationic groups in (C_6_H_5_N_2_)_2_SiF_6_ and (C_10_H_10_N_2_)SiF_6_ both exhibit ordered parallel arrangements under the guidance of inorganic rigid spherical [SiF_6_]^2^^−^ anionic groups (Figure 3b,d; Figure S3, Supporting Information). This optimal parallel structure is conducive to maximizing optical anisotropy and generating obvious birefringence. It cannot be ignored that the diprotonated [C_10_H_10_N_2_]^2+^ cation in (C_10_H_10_N_2_)SiF_6_ has more hydrogen bond formation positions compared to (C_6_H_5_N_2_)_2_SiF_6_. The N atoms on the opposite sides within its molecule undergo protonation and are connected in series with the SiF_6_ units, resulting in the (C_10_H_10_N_2_)SiF_6_ exhibiting a 3D structure. Although (C_10_H_10_N_2_)SiF_6_ has more N‐H···F hydrogen bond formation positions and its hydrogen bond interaction force is stronger (Figure 2e). Structural analysis shows (Figure 3) that the N‐H···F hydrogen bonds in compound (C_6_H_5_N_2_)_2_SiF_6_ are arranged completely parallel and in the same orientation. For the compound (C_10_H_10_N_2_)SiF_6_, there is a 57.57° dihedral angle between the two N‐H···F hydrogen bonds on each protonated 2,2′‐bipyridine group, and they are repeatedly arranged along the coordinate axes. The existence of this dihedral angle prevents the hydrogen bonds from forming a completely parallel arrangement, which is unfavorable for effectively enhancing generating excellent optical anisotropy. This also means that the improvement in birefringence performance from (C_6_H_5_N_2_)_2_SiF_6_ to (C_10_H_10_N_2_)SiF_6_ is not significant (0.505 to 0.583@546 nm). The increase in birefringence can mainly be attributed to the fact that the protonated [C_10_H_10_N_2_]^2+^ cation in (C_10_H_10_N_2_)SiF_6_ has a greater polarization anisotropy than the protonated [C_6_H_5_N_2_]^+^ cation in (C_6_H_5_N_2_)_2_SiF_6_ (110.1 vs 66.56, Figure 1a).

Hirshfeld surface analysis is often used to visualize intermolecular interactions, especially those involved in hydrogen bonds.^[^ ^54^, ^55^ ^]^ The Hirshfeld surface analysis of both compounds reveals distinct red spots, which correspond to regions of close intermolecular contact (Figure 2b,d). This also indicate the hydrogen bond interaction between the inorganic rigid SiF_6_ anion and the organic π‐conjugated cation, which is consistent with the structural features discussed earlier. The intermolecular hydrogen bonding in two compounds is a main factor in the crystal packing, which accounts for 62.6% and 37.5% of the Hirshfeld surface of these molecules in Figure 2c,e. Other intermolecular interactions such as C···C, C···N, and N···F contribute comparatively far less to crystal packing, as shown in Figure 2f,g (The 2D fingerprint spectra of other intermolecular interactions are detailed in (Figures S11 and S12, Supporting Information). Therefore, the packing motifs of the two molecules are dominated by N‐H···F hydrogen bonds.^[^ ^17^ ^]^

The infrared (IR) spectra (Figure S9, Supporting Information) of (C_6_H_5_N_2_)_2_SiF_6_ and (C_10_H_10_N_2_)SiF_6_ demonstrate their broad transparency range from 4000 to 400 cm^−1^,^[^ ^17^ ^]^ making them highly suitable for optical applications. In (C_6_H_5_N_2_)_2_SiF_6_, the stretching vibration of the characteristic C≡N bond in the protonated 4‐cyanopyridine cation is 2221 cm^−1^.^[^ ^32^ ^]^ The stretching vibrations of the Si‐F bond of the [SiF_6_]^2^^−^ anion group of two compounds could be observed at 520 cm^−1^ and 533/468 cm^−1^, respectively. The attribution of the remaining IR vibrational peaks of these two compounds is detailed in Table S5 (Supporting Information). These vibration peak distributions are similar to those of the previously reported organic‐inorganic hybrid fluorosilicates, confirming the structural integrity and functional group composition of the synthetic materials. The ultraviolet‐visible diffuse reflection spectra of (C_6_H_5_N_2_)_2_SiF_6_ and (C_10_H_10_N_2_)SiF_6_ are shown in Figure S8 (Supporting Information). Their UV absorption edges are 312 and 314 nm, respectively, indicating the ultraviolet transmission, and the corresponding optical band gaps are 3.79 and 3.61 eV, respectively. Furthermore, the transmission spectrum curve indicates that the cut‐off edge of (C_6_H_5_N_2_)_2_SiF_6_ is 306 nm (Figure S13, Supporting Information), which is in good agreement with the experimental result of the UV–visible–near‐IR diffuse reflectance spectroscopy.

**Figure 5:**
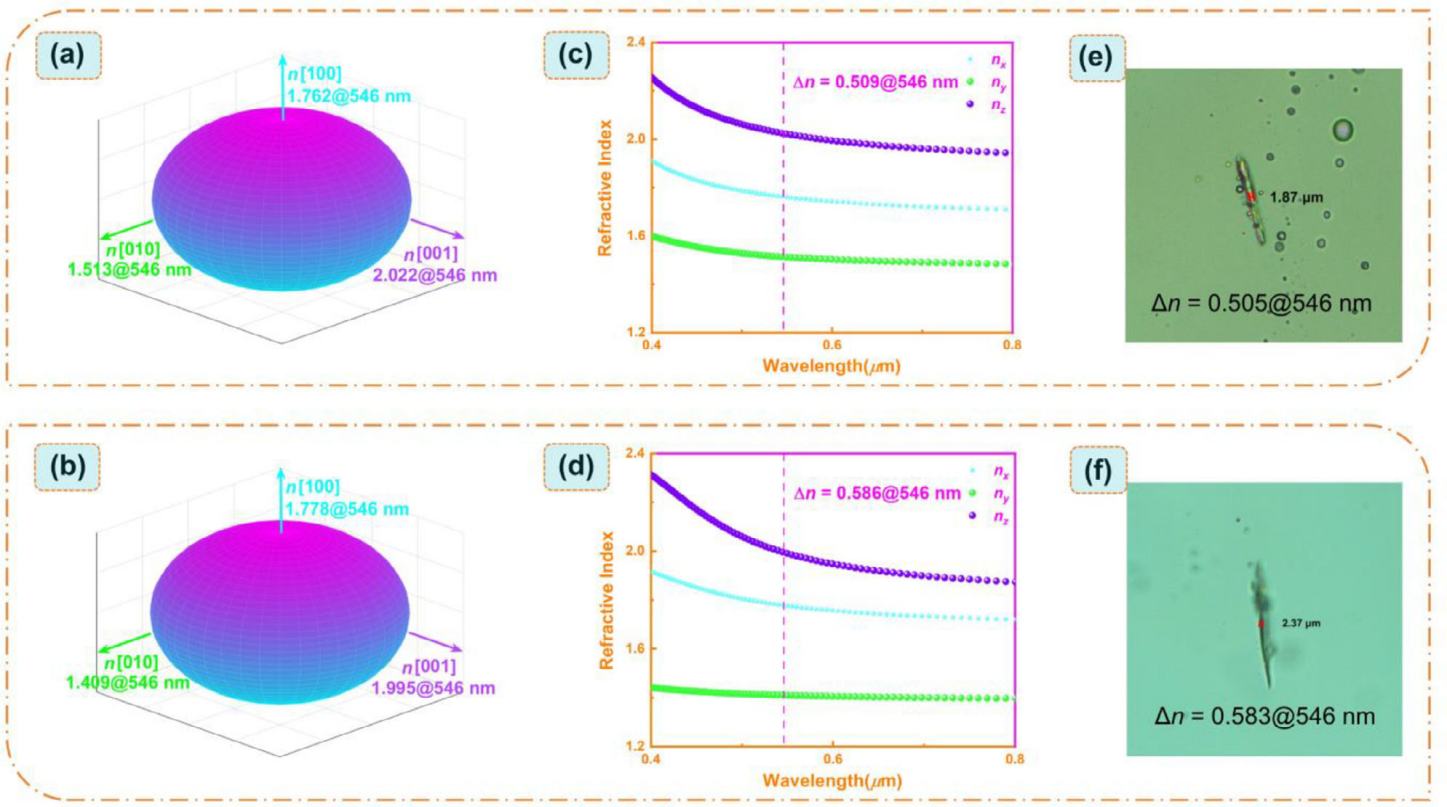
Triaxial ellipsoid of three principal refractive indices at 546 nm for a) (C_6_H_5_N_2_)_2_SiF_6_ and b) (C_10_H_10_N_2_)SiF_6_. The refractive index curve for c) (C_6_H_5_N_2_)_2_SiF_6_ and d) (C_10_H_10_N_2_)SiF_6_. The crystal thickness of e) (C_6_H_5_N_2_)_2_SiF_6_ and f) (C_10_H_10_N_2_)SiF_6_. The optical path difference (Δ*R*) is measured at 546 nm by using a polarizing microscope (Nikon LV1000) equipped with a Berek compensator.^[^ ^43^ ^]^

In order to evaluate the potential as birefringent crystals for these two compounds. The birefringences of (C_6_H_5_N_2_)_2_SiF_6_ and (C_10_H_10_N_2_)SiF_6_ were measured using thin slice crystals with selected compounds on an orthogonal polarizing microscope equipped with a Berek compensator. The wavelength of the incident light is 546 nm.^[^ ^5^ ^]^ After the polarized light passes through the crystal, birefringence occurs, forming two polarized lights with perpendicular polarization directions. The crystal thicknesses of these two compounds were obtained under a polarizing microscope as 1.87 µm and 2.37 µm, respectively (**Figure** **5**e,f). The crystal images of (C_6_H_5_N_2_)_2_SiF_6_ and (C_10_H_10_N_2_)SiF_6_ in their initial states and after complete extinction are detailed in Figure S10 (Supporting Information). The optical path differences (*R*) required for complete extinction from orthogonal polarization were determined to be 944.41 nm for (C_6_H_5_N_2_)_2_SiF_6_ and 1382.43 nm for (C_10_H_10_N_2_)SiF_6_. According to the formula *R* = Δ*n* × *d*, the birefringence values of these two compounds can be calculated as 0.505@546 nm and 0.583@546 nm, respectively. Notably, the experimental birefringence value of (C_10_H_10_N_2_)SiF_6_ (0.583@546 nm) is the highest among all reported fluorides containing the rigid group MF_6_ and also the largest in the fluorosilicate family, including (C_2_H_4_N_4_)_4_ZnSiF_6_·H_2_O (Δ*n*_exp._ = 0.282@546 nm), [C_10_H_8_NO_2_]_2_SiF_6_·H_2_O (Δ*n*_exp._ = 0.282@550 nm), RbGaF_3_(IO_3_) (Δ*n*_cal._ = 0.174@1064 nm), [GaF(H_2_O)][IO_3_F] (Δ*n*_cal._ = 0.166@532 nm), (C_3_N_6_H_7_)_2_SiF_6_·H_2_O (Δ*n*_cal._ = 0.152@550 nm), Pb_2_(SeO_3_)(SiF_6_) (Δ*n*_exp._ = 0.147@546 nm), (C_2_H_7_N_4_O)_2_SiF_6_ (Δ*n*_exp._ = 0.135@546 nm), and Li_2_KAl_2_F_9_ (Δ*n*_cal._ = 0.0056@1064 nm).^[^ ^56^, ^57^ ^]^ Likewise, it far exceeds the birefringent crystal materials of inorganic fluorides (Table S4, Supporting Information lists the information of some other related fluoride birefringent crystals). As shown in Figure 2a, the birefringent comparison of the currently reported crystal materials containing rigid inorganic groups is presented. It is worth noting that among all the fluoride birefringent crystals reported so far, only (C_12_H_8_N_2_)SbF_3_ has a greater experimental birefringence than (C_10_H_10_N_2_)SiF_6_ (0.583@546 nm),^[^ ^33^ ^]^ but (C_10_H_10_N_2_)SiF_6_ has a larger experimental band gap (3.61 vs 3.45 eV), making it an excellent overall performance birefringent material. Furthermore, the birefringence properties of these two compounds are superior to those of all current commercial birefringent crystals, including, MgF_2_ (0.012@546 nm), α‐BaB_2_O_4_ (0.1238@546 nm), CaCO_3_ (0.172@546 nm), and TiO_2_ (0.306@546 nm),^[^ ^4^, ^5^ ^]^ making them promising candidates for birefringent crystals.

The electronic band structure, density of states (DOS), and birefringence were assessed with the CASTEP package to provide a deeper insight into the structure‐property relation‐ship. The theoretical band gap of (C_6_H_5_N_2_)_2_SiF_6_ was calculated to be 3.48 eV, which is an indirect band gap. The theoretical energy gap of (C_10_H_10_N_2_)SiF_6_ is 3.00 eV, which is a direct band gap (Figure S8, Supporting Information). The difference between the calculated and experimental band gap can be attributed to the discontinuous nature of the GGA‐PBE's exchange correction function.^[^ ^45^ ^]^ Therefore, in order to accurately analyze its optical properties, we used scissors operators of 0.31 and 0.61 eV for (C_6_H_5_N_2_)_2_SiF_6_ and (C_10_H_10_N_2_)SiF_6_, respectively. The total and partial density of states of these compounds are shown in Figure S7 (Supporting Information). The orbit near Fermi energy plays a key role in optical properties because electrons jump from the top of the valence band to the bottom of the conduction band.

For (C_6_H_5_N_2_)_2_SiF_6_, the top of the valence band is mainly contributed by F‐2p, C‐2p and N‐2p orbitals, and the bottom of the conduction band is mainly composed of C‐2p and N‐2p orbitals hybridized with a small number of Si‐3p orbitals. In (C_10_H_10_N_2_)SiF_6_, the top of the valence band is mainly contributed by F‐2p and C‐2p orbitals, while the bottom of the conduction band is mainly composed of C‐2p and N‐2p orbitals. Observing the DOS diagram, it can be found that the charge transfer between the valence band and the conduction band is mainly determined by C, N and F atoms, indicating that organic groups and fluorine have a significant influence on its optical properties.^[^ ^56^ ^]^ For (C_6_H_5_N_2_)_2_SiF_6_, the overlapping of C and N orbitals in both the conduction band and the valence band also indicates the presence of a strong C≡N covalent bond in the protonated 4‐cyanopyridine cation. Furthermore, there is an overlap of F‐2P and N‐2p states in the valence band regions of these compounds, indicating that N‐H···F hydrogen bonds exist in both of these two compounds. It can be seen that their band gaps are mainly dominated by π‐conjugated cationic groups and F^−^ ions.

According to the first‐principles calculation results, it can be known that both (C_6_H_5_N_2_)_2_SiF_6_ and (C_10_H_10_N_2_)SiF_6_ have obvious linear optical properties (Figure 5c,d). (C_6_H_5_N_2_)_2_SiF_6_ and (C_10_H_10_N_2_)SiF_6_ crystallize respectively in the triclinic and monoclinic crystal systems, both belonging to the biaxial crystal system, and their refractive indices all satisfy the sequential relationship of *n_X_* > *n_Y_* > *n_Z_*. Specifically, for (C_6_H_5_N_2_)_2_SiF_6_ and (C_10_H_10_N_2_)SiF_6_, the sequence of their three refractive indices all satisfies the relationship of *n*[001] > *n*[100] > *n*[010]. To align with the standard crystal axis, we performed the conversions of *n*[001] → *n_X_*, *n*[100] → *n_Y_* and *n*[010] → *n_Z_*, respectively (Figure 5a,b). At a wavelength of 546 nm, the static refractive index is: for (C_6_H_5_N_2_)_2_SiF_6_, *n*[100] = 1.762, *n*[010] = 1.513, *n*[001] = 2.022; for (C_10_H_10_N_2_)SiF_6_, *n*[100] = 1.778, *n*[010] = 1.409, *n*[001] = 1.995. The corresponding theoretical birefringences values ($\Delta n_{max}^{cal.}$ = *n_X_* – *n_Z_*) are 0.509@546 nm and 0.586@546 nm, respectively. The theoretical calculated birefringence of these two compounds is in good agreement with the measured birefringences.

It is worth noting that (C_6_H_5_N_2_)_2_SiF_6_ and (C_10_H_10_N_2_)SiF_6_ have similar crystal structures, although the two six‐membered rings in the protonated 2,2′‐bipyridine cation have a dihedral angle of 36.38° rather than being coplanar. However, the organic π‐conjugated cations in both compounds undergo ordered arrangement under the guidance of rigid inorganic SiF_6_ units, that is, macroscopically, they both show parallel arrangements and spread in different directions (Figure 4d,e). This optimal arrangement helps to increase the birefringence. This is also the main reason why both compounds exhibit excellent optical anisotropy. We calculated the electron density difference diagrams of the two compounds (Figure 4c). By observing the distribution of the electron cloud, it can be found that the π‐conjugated [C_6_H_5_N_2_]^+^ and [C_10_H_10_N_2_]^2+^ cations both exhibit obvious π‐conjugated structural characteristics and have strong optical anisotropy. The contribution of their introduction to microscopic anisotropy is obvious at the microscopic level, which is conducive to generating large birefringence.^[^ ^26^ ^]^ In addition, the local HOMO and highest occupied molecular orbital (HOMO)–lowest unoccupied molecular orbital (LUMO) patterns of (C_6_H_5_N_2_)_2_SiF_6_ and (C_10_H_10_N_2_)SiF_6_ are shown in Figure 4a,b. For these two compounds, the HOMO is mainly consists of F‐2p orbitals, while LUMO is dominated by the π‐conjugated orbitals.^[^ ^4^ ^]^ Overall, the π‐conjugated [C_6_H_5_N_2_]^+^, [C_10_H_10_N_2_]^2+^ cations and fluorine atoms play a major role in their optical properties. Both cations [C_6_H_5_N_2_]^+^ and [C_10_H_10_N_2_]^2+^ selected in this work exhibit significant polarization anisotropy and relatively appropriate optical band gaps, surpassing most organic π‐conjugated groups such as [C_5_H_7_N_2_]^+^,^[^ ^32^ ^]^ [C_4_H_6_N_3_]^+^,^[^ ^58^ ^]^ [C_2_H_7_N_4_O]^+^,^[^ ^56^ ^]^ [C_3_N_3_O_3_],3^−[^ ^58^ ^]^ [CH_7_N_4_]^+^,^[^ ^17^ ^]^ and [B_3_O_6_], ^3^^−[^ ^46^ ^]^ calculated using DFT implemented by the Gaussian16 package at the B3LYP/6‐31G level (Figure 1a). And both of these π‐conjugated cations have coplanar hydrogen atoms, which can form hydrogen bonds with N/O/F atoms, thereby promoting the constraints of spatial freedom within the module. Structural analysis also confirm this point.^[^ ^17^ ^]^ We also calculated the functional unit densities in (C_6_H_5_N_2_)_2_SiF_6_ and (C_10_H_10_N_2_)SiF_6_ (Table S8, Supporting Information). The densities of the inorganic rigid [SiF_6_]^2^^−^ anion groups in the two compounds are similar, while the density of the organic groups in (C_10_H_10_N_2_)SiF_6_ is significantly lower than that in (C_6_H_5_N_2_)_2_SiF_6_, but it shows greater birefringence (0.583@546 nm). Mainly because the protonated π‐conjugated [C_10_H_10_N_2_]^2+^ cation has a higher polarizability anisotropy than the protonated π‐conjugated [C_6_H_5_N_2_]^+^ cation, (C_10_H_10_N_2_)SiF_6_ has more superior linear optical properties.^[^ ^42^ ^]^ Distinctly, the introduction of planar π‐conjugated groups is a very effective strategy for achieving excellent birefringence. Owing to π‐conjugated molecular orbital interactions, π‐conjugated systems exhibit high polarizability anisotropy and induce large birefringence in compounds, especially through units with six‐membered ring geometries.

Although the birefringence of (C_10_H_10_N_2_)SiF_6_ has been improved compared to (C_6_H_5_N_2_)_2_SiF_6_, there is still the possibility of a breakthrough in the birefringence of this system. In (C_10_H_10_N_2_)SiF_6_, although the protonated [C_10_H_10_N_2_]^2+^ cations are arranged in an ordered manner,^[^ ^59^ ^]^ there is a dihedral angle (θ = 36.38°) between its two six‐membered rings (Figure 4e), which also makes the crystal structure of (C_10_H_10_N_2_)SiF_6_ not form the most perfect parallel arrangement like that of (C_6_H_5_N_2_)_2_SiF_6_, but only the single six‐membered rings facing the same direction are arranged parallel to each other. In the subsequent work, we will continuously attempt to use organic π‐conjugated ligands with higher polarizability anisotropy and multiple coplanar hexagonal rings simultaneously to synthesize organic‐inorganic hybrid birefringent materials with more excellent comprehensive performance.

## 3 Conclusion

In conclusions, two organic‐inorganic hybrid fluorosilicate birefringent crystals, (C_6_H_5_N_2_)_2_SiF_6_ and (C_10_H_10_N_2_)SiF_6_, were sequentially synthesized the aqueous solution evaporation method. Structural analysis reveals that in these two compounds, both the π‐conjugated 4‐cyanopyridine cation and the 2,2′‐bipyridine cation have formed hydrogen bonds with the rigid inorganic [SiF_6_]^2^^−^ anionic group. Moreover, under the guidance of the SiF_6_ unit, both organic ligands exhibit parallel alignment in the multidirectional upward direction. This optimal arrangement endows them with excellent linear optical properties. This work enriches the fluorosilicate family. (C_10_H_10_N_2_)SiF_6_ has the largest birefringence among the current fluorosilicates and surpasses all current inorganic fluoride crystals and all commercial birefringent crystals. First‐principles calculations show that the introduction of π‐conjugated [C_6_H_5_N_2_]^+^ and [C_10_H_10_N_2_]^2+^ cations is the main cause of the increase in birefringence, and the influence of organic groups on the structure and optical properties of the compound is also demonstrated. This work not only provides two new types of birefringent crystals, but also offers new ideas for exploring high‐performance fluoride birefringent crystal materials. In the future, we will continue to explore high‐performance birefringent crystals by introducing π‐conjugated organic groups.

## Conflict of Interest

The authors declare no conflict of interest.

## Supporting information



Supporting Information

## Data Availability

The data that support the findings of this study are available in the supplementary material of this article.

## References

[1] L. H. Nicholls , F. J. Rodríguez‐Fortuño , M. E. Nasir , R. M. Córdova‐Castro , N. Olivier , G. A. Wurtz , A. V. Zayats , Nat. Photonics 2017, 11, 628.

[2] S. Y. Niu , G. Joe , H. Zhao , Y. C. Zhou , T. Orvis , H. X. Huyan , J. Salman , K. Mahalingam , B. Urwin , J. B. Wu , Y. Liu , T. E. Tiwald , S. B. Cronin , B. M. Howe , M. Mecklenburg , R. Haiges , D. J. Singh , H. Wang , M. A. Kats , J. Ravichandran , Nat. Photonics 2018, 12, 392.

[3] L. Wu , S. Patankar , T. Morimoto , N. L. Nair , E. Thewalt , A. Little , J. G. Analytis , J. E. Moore , J. Orenstein , Nat. Phys. 2017, 13, 350.

[4] M. Arif , X. Liu , H. W. Jia , Z. H. Yang , X. L. Hou , S. L. Pan , Small 2025, 21, 2500633. 10.1002/smll.202500633 40405616

[5] J. Chen , M. B. Xu , H. Y. Wu , J. Y. Wu , K. Z. Du , Angew. Chem., Int. Ed. 2024, 63, 202411503. 10.1002/anie.202411503 38985723

[6] D. Y. Dou , C. Wei , B. B. Zhang , D. Q. Yang , Y. Wang , Angew. Chem., Int. Ed. 2025, 64, 202504761. 10.1002/anie.202504761 40133208

[7] W. Lu , Z. L. Gao , X. T. Liu , X. X. Tian , Q. Wu , C. G. Li , Y. X. Sun , Y. Liu , X. T. Tao , J. Am. Chem. Soc. 2018, 140, 13089. 30212626 10.1021/jacs.8b08803

[8] X. L. Chen , B. B. Zhang , F. F. Zhang , Y. Wang , M. Zhang , Z. H. Yang , K. R. Poeppelmeier , S. L. Pan , J. Am. Chem. Soc. 2018, 140, 16311. 30418021 10.1021/jacs.8b10009

[9] I. A. Kruglov , L. A. Bereznikova , C. W. Xie , D. D. Chu , K. Li , E. Tikhonov , A. Tudi , A. Mazitov , M. Zhang , S. L. Pan , Z. H. Yang , Sci. China Mater. 2024, 67, 3941.

[10] H. T. Qiu , R. An , C. Cui , Z. Li , J. F. Yang , X. C. Wang , X. L. Hou , J. J. Li , J. J. Lu , J. Sun , Z. H. Yang , S. L. Pan , M. Mutailipu , Angew. Chem., Int. Ed. 2025, 64, 202507171. 10.1002/anie.202507171 40275034

[11] Z. Q. Chen , C. X. Li , X. Y. Wu , J. J. Lu , Z. H. Yang , X. L. Hou , M. Mutailipu , Aggregate 2025, 70134.

[12] M. J. Li , X. Zhang , Z. Y. Xiong , Y. Q. Li , Y. Zhou , X. Chen , Y. P. Song , M. C. Hong , J. H. Luo , S. G. Zhao , Angew. Chem., Int. Ed. 2022, 61, 202211151. 10.1002/anie.202211151 36018802

[13] Q. T. Xu , Z. Y. Wang , Y. Zhou , W. Q. Huang , Y. P. Song , J. Y. Zheng , Y. Q. Li , L. X. Hou , J. H. Luo , S. G. Zhao , Adv. Funct. Mater. 2024, 35, 2417431.

[14] J. H. Wu , C. L. Hu , Y. F. Li , J. G. Mao , F. Kong , Chem. Sci. 2024, 15, 8071. 38817564 10.1039/d4sc01716a PMC11134327

[15] H. T. Tian , C. S. Lin , Y. Q. Zhou , X. Zhao , H. X. Fan , T. Yan , N. Ye , M. Luo , Angew. Chem., Int. Ed. 2023, 62, 202304858. 10.1002/anie.202304858 37218024

[16] Z. Y. Wang , X. Chen , Y. P. Song , Z. P. Du , Y. Zhou , M. J. Li , W. Q. Huang , Q. T. Xu , Y. Q. Li , S. G. Zhao , J. H. Luo , Angew. Chem., Int. Ed. 2023, 62, 202311086. 10.1002/anie.202311086 37766424

[17] H. Zhou , M. Cheng , D. D. Chu , X. Liu , R. An , S. L. Pan , Z. H. Yang , Angew. Chem., Int. Ed. 2025, 64, 202413680. 10.1002/anie.202413680 39143747

[18] Y. Zhou , Z. F. Guo , H. G. Gu , Y. Q. Li , Y. P. Song , S. Y. Liu , M. C. Hong , S. G. Zhao , J. H. Luo , Nat. Photonics. 2024, 18, 922.

[19] J. B. Wang , M. M. Zhu , Y. Q. Chu , J. D. Tian , L. L. Liu , B. B. Zhang , P. S. Halasyamani , Small 2024, 20, 2308884. 10.1002/smll.202308884 38098344

[20] Z. P. Du , X. Y. Song , W. Liu , Z. Y. Wang , H. Y. Sha , Q. T. Xu , Z. Yang , Y. Q. Li , J. H. Luo , S. G. Zhao , Sci. Bull. 2024, 69, 2205. 10.1016/j.scib.2024.04.006 38599957

[21] W. Liu , Z. P. Du , B. Ahmed , Y. Q. Zhao , J. Y. Zheng , X. Y. Song , Z. Y. Wang , X. J. Kuang , J. H. Luo , S. G. Zhao , Adv. Optical Mater. 2024, 12, 2400474.

[22] J. H. Wu , C. L. Hu , T. Y. Jiang , J. G. Mao , F. Z. Kong , J. Am. Chem. Soc. 2023, 145, 24416. 37881867 10.1021/jacs.3c09566

[23] X. H. Dong , L. Huang , H. M. Zeng , Z. H. Lin , K. M. Ok , G. H. Zou , Angew. Chem., Int. Ed. 2025, 64, 202509917. 10.1002/anie.202509917 40539531

[24] Z. Y. Bai , J. Y. Lee , C. L. Hu , G. H. Zou , K. M. Ok , Chem. Sci. 2024, 15, 6572. 38699253 10.1039/d4sc01353k PMC11062127

[25] A. Tudi , Z. J. Li , C. W. Xie , T. Baiheti , E. V. Tikhonov , F. F. Zhang , S. L. Pan , Z. H. Yang , Adv. Funct. Mater. 2024, 34, 2409716.

[26] Y. G. Shen , M. L. Ding , G. Chen , Y. J. Luo , S. G. Zhao , J. H. Luo , Small 2024, 20, 2400549. 10.1002/smll.202400549 38726954

[27] Y. Q. Zhang , Q. R. Ding , Y. Q. Li , X. Y. Song , W. Q. Huang , Y. Zhou , Q. P. Shao , Z. Y. Bai , S. G. Zhao , J. H. Luo , Laser Photonics Rev. 2025, 19, 2500368.

[28] Y. Y. Sun , Y. Q. Li , X. Y. Song , S. G. Zhao , T. H. Zhou , J. Zhang , Adv. Funct. Mater. 2025, 35, 2413107.

[29] J. Y. Zheng , X. Y. Song , Y. R. Wu , Y. L. Lian , Y. Q. Li , Q. T. Xu , Y. Zhou , Z. Y. Wang , L. Wang , J. H. Luo , S. G. Zhao , Adv. Funct. Mater. 2024, 34, 2403843.

[30] Q. T. Xu , Z. Y. Wang , Y. Zhou , W. Q. Huang , Y. P. Song , J. Y. Zheng , Y. Q. Li , L. X. Hou , J. H. Luo , S. G. Zhao , Adv. Funct. Mater. 2025, 35, 2417431.

[31] Y. Q. Li , X. Y. Song , B. Chen , Y. P. Song , W. Q. Huang , J. H. Luo , S. G. Zhao , Mater. Today 2025, 87, 29.

[32] R. L. Tang , D. X. Yang , L. Ma , Y. L. Lv , W. L. Liu , S. P. Guo , Adv. Optical Mater. 2024, 13, 2403044.

[33] P. Zhang , X. H. Dong , L. Huang , Z. E. Lin , Y. Q. Zhou , G. H. Zou , Angew. Chem., Int. Ed. 2025, 64, 202424756.

[34] P. Zhang , X. Mao , X. H. Dong , L. Huang , L. L. Cao , D. J. Gao , G. H. Zou , Chin. Chem. Lett. 2024, 35, 109235.

[35] Q. T. Xu , W. Q. Huang , H. Wang , Y. Q. Li , Y. Zhou , L. X. Hou , S. G. Zhao , J. H. Luo , Small 2023, 19, 2304333. 10.1002/smll.202304333 37616508

[36] Z. C. Wang , Q. Y. Liu , Z. H. Yang , X. L. Hou , M. Q. Gai , Small 2025, 21, 2502594. 10.1002/smll.202502594 40191875

[37] Z. Y. Bai , J. H. Lee , H. W. Kim , C.‐L. Hu , K.‐M. Ok , Small 2023, 19, 2301756. 10.1002/smll.202301756 36970809

[38] Y. L. Zhu , J. Gou , C. Yang , Q. W. Zhu , Y. Xiong , Q. Wu , Angew. Chem., Int. Ed. 2025, 64, 202509290. 10.1002/anie.202509290 40401594

[39] W. Q. Huang , X. L. Wu , B. Ahmed , Y. Q. Li , Y. Zhou , H. Wang , Y. P. Song , X. J. Kuang , J. H. Luo , S. G. Zhao , Inorg. Chem. Front. 2023, 10, 2039.

[40] Z. Y. Bai , K. M. Ok , Angew. Chem., Int. Ed. 2024, 63, 202315311.

[41] C. Yang , Y. W. Kang , X. F. Wang , J. Gou , Y. Xiong , L. Chen , Q. Wu , Chem. Sci. 2024, 15, 15725. 39263656 10.1039/d4sc04476b PMC11382538

[42] L. Qi , X. X. Jiang , K. N. Duanmu , C. Wu , Z. S. Lin , Z. P. Huang , M. G. Humphrey , C. Zhang , J. Am. Chem. Soc. 2024, 146, 9975. 38466811 10.1021/jacs.4c00666

[43] M. B. Xu , Q. Q. Chen , B. X. Li , K. Z. Du , J. Chen , Chin. Chem. Lett. 2025, 36, 110513.

[44] Z. Y. Bai , K. M. Ok , Small 2024, 20, 2311391. 10.1002/smll.202311391 38233208

[45] J. C. Lu , K. M. Ok , Chem. Sci. 2025, 16, 4703. 39935502 10.1039/d5sc00112a PMC11808400

[46] H. P. Wu , Z. J. Wei , Z. G. Hu , J. Y. Wang , Y. C. Wu , H. W. Yu , Angew. Chem., Int. Ed. 2024, 63, 202406318. 10.1002/anie.202406318 38715104

[47] L. Qiu , L. Ma , W. L. Liu , Y. Y. Sun , R. L. Tang , Adv. Optical Mater. 2025, 13, 2500149.

[48] X. H. Dong , L. Huang , C. F. Hu , H. M. Zeng , Z. E. Lin , X. Wang , K. M. OK , G. H. Zou , Angew. Chem., Int. Ed. 2019, 58, 6528. 10.1002/anie.201900637 30805990

[49] Y. L. Deng , L. Huang , X. H. Dong , L. Wang , K. M. OK , H. M. Zeng , Z. E. Lin , G. H. Zou , Angew. Chem., Int. Ed. 2020, 59, 21151. 10.1002/anie.202009441 32745331

[50] Y. Tian , W. Zeng , X. H. Dong , L. Huang , Y. Q. Zhou , H. M. Zeng , Z. E. Lin , G. H. Zou , Angew. Chem., Int. Ed. 2024, 63, 202409093. 10.1002/anie.202409093 38850113

[51] X. H. Dong , L. Huang , G. H. Zou , Acc. Chem. Res. 2025, 58, 150. 39667023 10.1021/acs.accounts.4c00704

[52] V. O. Gelmboldt , E. V. Ganin , M. M. Botoshansky , V. Y Anisimov , O. V. Prodan , V. C. Kravtsov , M. S. Fonari , J. Fluorine Chem. 2014, 160, 57.

[53] T. Ohmi , S. Kundu , H. Y. Ye , W. Zhou , K. Ogawa , Y. Haruta , A. M. Navarrete‐López , T. Yamamoto , M. I. Saidaminov , Inorg. Chem. 2025, 64, 12342. 40479616 10.1021/acs.inorgchem.5c01798

[54] P. R. Spackman , M. J. Turner , J. J. McKinnon , S. K. Wolff , D. J. Grimwood , D. Jayatilaka , M. A. Spackman , J. Appl. Crystallogr. 2021, 54, 1006. 34188619 10.1107/S1600576721002910 PMC8202033

[55] M. A. Spackman , D. Jayatilakaa , CrystEngComm 2009, 11, 19.

[56] L. Ma , Y. L. Lv , B. W. Miao , G. R. Zhu , W. L. Liu , S. P. Guo , R. L. Tang , Inorg. Chem. Front. 2025, 12, 4804.

[57] P. F. Li , C. L. Hu , J. G. Mao , F. Kong , Chem. Sci. 2024, 15, 7104. 38756790 10.1039/d4sc01376j PMC11095375

[58] Y. L. Lv , L. Ma , G. R. Zhu , B. W. Miao , W. L. Liu , S. P. Guo , R. L. Tang , Inorg. Chem. Front. 2025, 12, 5046.

[59] Z. C. Wu , S. P. Guo , Coord. Chem. Rev. 2025, 542, 216866.

