# Supplementary material for: Exceptional Enhancement of Optical Anisotropy Achieved via the Strategy of Combining Rigid Groups with High Symmetry and π‐Conjugated Organic Groups in Hybrid Fluorides
Source: Adv Sci (Weinh). 2025 Oct 6;12(48):e15170. doi: 10.1002/advs.202515170 (PMC12752605; doi:10.1002/advs.202515170)
Supplement: Supplementary file 1 — Supporting Information [file ADVS-12-e15170-s001.docx]

***Supporting Information***

**Exceptional Enhancement of Optical Anisotropy Achieved via the Strategy of Combining Rigid Groups with High Symmetry and π-Conjugated Organic Groups in Hybrid Fluorides**

Ru-Ling Tang,*^[a]^ Bing-Wei Miao,^[a]^ Guo-Ren Zhu, ^[a]^ Wenlong Liu, ^[a]^ and Sheng-Ping Guo*^[a,b]^

1. Ru-Ling Tang, Bing-Wei Miao, Guo-Ren Zhu, Wenlong Liu, and Sheng-Ping Guo
   School of Chemistry and Chemical Engineering
   Yangzhou University, Yangzhou 225002, P. R. China.
   E-mail: rltang@yzu.edu.cn, spguo@yzu.edu.cn
2. Sheng-Ping Guo

School of Materials and Energy, Yunnan Key Laboratory of Electromagnetic Materials and Devices

Yunnan University, Kunming 650500, P. R. China

*Corresponding authors: rltang@yzu.edu.cn; spguo@yzu.edu.cn.*

**Supporting Information Index**

1. **Experimental Section.**
2. Reagents.
3. Syntheses of (C_6_H_5_N_2_)_2_SiF_6_ and (C_10_H_10_N_2_)SiF_6_.
4. Single-Crystal Structure Determination.
5. Energy-Dispersive X-ray Spectroscopy (EDS).
6. Powder X-Ray Diffraction (PXRD) Analyses.
7. UV-vis-NIR Diffuse Reflectance and Infrared (IR) Spectroscopies.
8. Thermal Stability Analyses.
9. Birefringence Measurements.
10. Details of Theoretical Calculations.
11. **Tables and Figures**
12. **Table S1.** Refinement of crystal data and structural parameters of (C_6_H_5_N_2_)_2_SiF_6_ and (C_10_H_10_N_2_)SiF_6_.
13. **Table S2.** Selected bond lengths (Å) and bond angles (°) for (C_6_H_5_N_2_)_2_SiF_6_ and (C_10_H_10_N_2_)SiF_6_.
14. **Table S3.** Atomic coordinates (Å × 10^4^) and equivalent isotropic displacement parameters (*U*eq^a^, Å^2^ × 10^3^) for (C_6_H_5_N_2_)_2_SiF_6_ and (C_10_H_10_N_2_)SiF_6_.
15. **Table S4.** Related fluoride birefringent crystals.
16. **Table S5.** The assignment of the IR absorption peaks for (C_6_H_5_N_2_)_2_SiF_6_ and (C_10_H_10_N_2_)SiF_6_.
17. **Table S6.** Hydrogen atom coordinates (Å × 10^4^) and isotropic displacement parameters (Å^2^ × 10^3^) for (C_6_H_5_N_2_)_2_SiF_6_ and (C_10_H_10_N_2_)SiF_6_.
18. **Table S7.** Hydrogen bonds for (C_6_H_5_N_2_)_2_SiF_6_ and (C_10_H_10_N_2_)SiF_6_.
19. **Table S8.** Functional unit densities of (C_6_H_5_N_2_)_2_SiF_6_ and (C_10_H_10_N_2_)SiF_6_.
20. **Figure S1.** Powder X-ray diffraction pattern of (C_6_H_5_N_2_)_2_SiF_6_ and (C_10_H_10_N_2_)SiF_6_.
21. **Figure S2.** Hydrogen bond display diagrams in (C_6_H_5_N_2_)_2_SiF_6_ and (C_10_H_10_N_2_)SiF_6._
22. **Figure S3.** The parallel arrangement and planar spacing of the [SiF_6_]^2-^ anion groups in (C_6_H_5_N_2_)_2_SiF_6_ and (C_10_H_10_N_2_)SiF_6_.
23. **Figure S4.** EDS images for (C_6_H_5_N_2_)_2_SiF_6_ and (C_10_H_10_N_2_)SiF_6_.
24. **Figure S5.** TG-DTA curves of (C_6_H_5_N_2_)_2_SiF_6_ and (C_10_H_10_N_2_)SiF_6_.
25. **Figure S6**. Calculated band structures of (C_6_H_5_N_2_)_2_SiF_6_ and (C_10_H_10_N_2_)SiF_6._
26. **Figure S7**. Density of states (DOS) of (C_6_H_5_N_2_)_2_SiF_6_ and (C_10_H_10_N_2_)SiF_6_. The fermi level is set at 0 eV.
27. **Figure S8.** The UV-vis diffuse reflectance spectra and optical bandgaps of (C_6_H_5_N_2_)_2_SiF_6_ and (C_10_H_10_N_2_)SiF_6_.
28. **Figure S9.** IR spectra of (C_6_H_5_N_2_)_2_SiF_6_ and (C_10_H_10_N_2_)SiF_6_.
29. **Figure S10.** Crystal photos of (C_6_H_5_N_2_)_2_SiF_6_ and (C_10_H_10_N_2_)SiF_6_ in their initial state and crystal photos after complete extinction.
30. **Figure S11.** 2D fingerprint plots for overall interactions and the rest individual interactions of atom types in crystal packing of (C_6_H_5_N_2_)_2_SiF_6_.
31. **Figure S12.** 2D fingerprint plots for overall interactions and the rest individual interactions of atom types in crystal packing of (C_10_H_10_N_2_)SiF_6_.
32. **Figure S13.** UV–Vis–NIR transmission spectrum of (C_6_H_5_N_2_)_2_SiF_6_.

EXPERIMENTAL SECTION

**Reagents.** All reagents include 4-cyanopyridine (damas, 4-C_6_H_4_N_2_, 99%), 2,2'-Dipyridyl (damas, C_10_H_8_N_2_, 99%) and fluorosilicic acid (H_2_SiF_6_, Adamas, 30% aqueous solution), none of which require further purification. (Caution! Fluorosilicic acid is corrosive. It must be handled with extreme caution, using the appropriate protective equipment and training.)

**Syntheses of (C_6_H_5_N_2_)_2_SiF_6_ and (C_10_H_10_N_2_)SiF_6_.** The crystals of (C_6_H_5_N_2_)_2_SiF_6_ and (C_10_H_10_N_2_)SiF_6_ were prepared by a simple aqueous solution evaporation method. Accurately weigh the raw materials C_6_H_4_N_2_ (0.1067g, 1mmol) and C_10_H_8_N_2_ (0.1562g, 1mmol) respectively into two plastic cups, then, add H_2_SiF_6_ (0.5 mL) and deionized water (5 mL) to both of these plastic cups and then seal them. We stir the mixed solutions respectively with a magnetic stirrer for 25 minutes until they are completely dissolved, and then place the obtained clear solutions in an open-air dust-free environment to evaporate slowly. About 12 days later, colorless crystals could be observed in both plastic cups, which were (C_6_H_5_N_2_)_2_SiF_6_ and (C_10_H_10_N_2_)SiF_6_, respectively.

**Single-Crystal Structure Determination.** Clean single crystals of (C_6_H_5_N_2_)_2_SiF_6_ and (C_10_H_10_N_2_)SiF_6_ were selected for single-crystal XRD data collection on the Bruker D8 QUEST diffractometer equipped with a CCD detector (Mo K*α* radiation, *λ* = 0.71073 Å) at 296(2) K. Direct Method of SHELXTL program package was chosen to solve the structure, and full-matrix least-squares technique was used to refine all atoms. Crystal data,^[1,2]^ cell parameters, selected bond lengths and bond angles for (C_6_H_5_N_2_)_2_SiF_6_ and (C_10_H_10_N_2_)SiF_6_ are summarized in Tables S1‒S3. The single-crystal structure data of (C_6_H_5_N_2_)_2_SiF_6_ and (C_10_H_10_N_2_)SiF_6_ was also deposited with the CCDC number of 2468199 and 2468200.

**Energy-Dispersive Spectroscopy.** EDS analysis was performed on several selected crystals using a Bruker quantum dispersive X-ray spectroscope, and the result indicates the existence of elements Si, C, N and F exist in the crystals, with the ratios close to those determined from crystal structure analysis.

**Powder X-Ray Diffraction Analysis.** The blocky polycrystalline samples of (C_6_H_5_N_2_)_2_SiF_6_ and (C_10_H_10_N_2_)SiF_6_ were ground into powder and then used for powder XRD testing with an automated Bruker D8 X-ray diffractometer at room temperature. The test range was set from 10 to 70º with the scan step width of 0.02º. The result indicates that the experimental XRD pattern is identical with the simulated one converted from the cif document on Mercury program, which implies the purity of the obtained samples is high enough.

**UV-vis-NIR Diffuse Reflectance and Infrared (IR) Spectroscopies.** The UV-vis-NIR diffuse reflectance spectra were carried out on a Carry 5000 UV-vis-NIR spectrometer from 200-800 nm. Pure barium sulfate powder was used as a reference during the test.^[3]^ The IR spectra were collected on a Fourier transform IR spectrometer in the range of 4000-400 cm with pure KBr powder as background.

**Thermogravimetric Analyses.** The thermogravimetric analyses (TGA) were performed from 20 to 1000 ℃ with the rate of 10 ℃/min on the ground powder samples of (C_6_H_5_N_2_)_2_SiF_6_ and (C_10_H_10_N_2_)SiF_6_, with a Netzsch STA449F3 simultaneous analyzer and N_2_ as protective gas.

**Birefringence Measurements.** The optical path differences of (C_6_H_5_N_2_)_2_SiF_6_ and (C_10_H_10_N_2_)SiF_6_ were characterized using a polarizing microscope (Nikon LV1000) equipped with a Berek compensator. The incident wavelength of the light source is 546 nm.^[4]^ Under orthogonal polarization, two polarized light beams pass through the crystal at different speeds, and an interference color phenomenon occurs after passing through the analyzer. Small and thin crystals were selected during the measurement process. The formula of "R = (|Ne-No|) × T = Δ*n* × T" was used to calculate birefringence, in which R means optical path difference, Δ*n* represents birefringence, and T refers to the thickness of the crystal.

**Theoretical calculations.** The structure optimization and first-principles calculations for (C_6_H_5_N_2_)_2_SiF_6_ and (C_10_H_10_N_2_)SiF_6_ were performed by CASTEP on a plane-wave pseudopotential total energy package based density functional theory (DFT).^[5]^ The functional developed by Perdew-Burke-Ernzerhof (PBE) functional within the generalized gradient approximation (GGA) form was adopted to describe the exchange correlation energy.^[6]^ The ultrasoft pseudopotentials were used to model the effective interaction between atom cores and valence electrons.^[7]^ Based on this method, the band structures and density of states (DOS) of (C_6_H_5_N_2_)_2_SiF_6_ and (C_10_H_10_N_2_)SiF_6_ were calculated using the structural patterns obtained from SXRD analysis. The following orbital electrons were chosen as valence electrons: H 1*s*^1^, C 2*s*^2^2*p*^2^, N 2*s*^2^2*p*^3^, Si 3*s*^2^3*p*^2^, and F 2*s*^2^2*p*^5^. The cutoff energy of (C_6_H_5_N_2_)_2_SiF_6_ was set as 370 eV, with Monkhorst−Pack scheme set as 2 × 2 × 2 in Brillouin zone. The cutoff energy of (C_10_H_10_N_2_)SiF_6_ was set as 370 eV, with Monkhorst−Pack scheme set as 1 × 2 × 1 in Brillouin zone.^[8]^ The linear optical properties were examined based on the dielectric function ε(ω) = ε_1_(ω) + iε_2_(ω). The imaginary part of dielectric function ε_2_ can be calculated based on the electronic structures and the real part is obtained by the Kramers-Kronig transformation, accordingly the refractive indices and the birefringence (Δ*n*) can be calculated. The frequency-dependent refractive indices were calculated to demonstrate the validity of birefringence measurements.^[9,10]^ In addition, the polarizability anisotropy of the π-conjugated group, the HOMO-LUMO gap and the intramolecular local HOMO-LUMO orbital plots were all calculated using DFT implemented by the Gaussian16 package at the B3LYP/6-31G level.^[3]^

**TABLES AND FIGURES**

**Table S1.** Crystal data and structure refinement parameters for (C_6_H_5_N_2_)_2_SiF_6_ and (C_10_H_10_N_2_)SiF_6_.

| **Empirical formula** | **(C_6_H_5_N_2_)_2_SiF_6_** | **(C_10_H_10_N_2_)SiF_6_** |
| --- | --- | --- |
| Formula weight | 352.33 | 300.29 |
| Temperature/K | 296 | 296 |
| Crystal system | triclinic | monoclinic |
| Space group | *P*$\bar{1}$ | *C*2*/c* |
| *a*/Å | 7.2153(6) | 17.9015(19) |
| *b*/Å | 7.3952(6) | 6.6001(7) |
| *c*/Å | 7.8907(7) | 12.386(3) |
| *α*/° | 79.296(5) | 90 |
| *β*/° | 63.583(4) | 131.249(2) |
| *γ*/° | 66.275(4) | 90 |
| Volume/Å^3^ | 345.19(5) | 1100.3(3) |
| *Z* | 1 | 4 |
| *ρ*_calc_ g/cm^3^ | 1.695 | 1.813 |
| *μ*/mm^-1^ | 0.244 | 0.283 |
| *F*(000) | 178.0 | 608.0 |
| Radiation | MoK*α* (*λ* = 0.71073) | MoK*α* (*λ* = 0.71073) |
| 2*Θ* range for data collection/° | 5.766 to 50.718 | 6.054 to 50.732 |
| Index ranges | -8 ≤ h ≤ 8, -8 ≤ k ≤ 8, -9 ≤ l ≤ 9 | -18 ≤ h ≤ 21, -7 ≤ k ≤ 7, -14 ≤ l ≤ 14 |
| Reflections collected | 5613 | 4763 |
| Independent reflections | 1269 [*R*_int_ = 0.028, *R*_sigma_ = 0.0234] | 1003 [*R*_int_ = 0.0688, *R*_sigma_ = 0.0635] |
| Data/restraints/parameters | 1269/0/107 | 1003/0/89 |
| Goodness-of-fit on F^2^ | 1.043 | 1.009 |
| Final R indexes [I ≥ 2σ (I)] ^a,b^ | R_1_ = 0.0385, wR_2_ = 0.0868 | R_1_ = 0.0477, wR_2_ = 0.0821 |
| Final R indexes [all data]^a,b^ | R_1_ = 0.0493, wR_2_ = 0.0932 | R_1_ = 0.0914, wR_2_ = 0.0951 |
| Largest diff. peak/hole (e Å^–3^) | 0.35/-0.25 | 0.27/-0.25 |

*^a^R*_1_ = ∑||*F*_o_| − |*F*_c_||/∑|*F*_o_| and *^b^w*R_2_ = [*w* (*F*_o_^2^ − *F*_c_^2^)^2^/*wF*_o_^4^]^1/2^ for *F*_o_^2^> 2σ (*F*_o_^2^)

**Table S2.** Selected bond lengths (Å) and bond angles (°) for (C_6_H_5_N_2_)_2_SiF_6_ and (C_10_H_10_N_2_)SiF_6_.

| **Bond length (Å) for (C_6_H_5_N_2_)_2_SiF_6_** | | | |
| --- | --- | --- | --- |
| Si(1)－F(1)^1^ | 1.6654(13) | N(2)－C(3) | 1.130(3) |
| Si(1)－F(1) | 1.6654(13) | N(1)－C(5) | 1.332(3) |
| Si(1)－F(2)^1^ | 1.6959(13) | C(1)－C(4) | 1.379(3) |
| Si(1)－F(2) | 1.6959(13) | C(1)－C(6) | 1.388(3) |
| Si(1)－F(3) | 1.6642(13) | C(1)－C(3) | 1.451(3) |
| Si(1)－F(3)^1^ | 1.6642(13) | C(4)－C(2) | 1.370(3) |
| N(1)－C(2) | 1.332(3) | C(6)－C(5) | 1.369(3) |
|  |  |  |  |
|  |  |  |  |
|  |  |  |  |
|  |  |  |  |
|  |  |  |  |
|  |  |  |  |
| **Bond length (Å) for (C_10_H_10_N_2_)SiF_6_** | | | |
| Si(1)－F(1) | 1.6700(16) | N(1)－C(2) | 1.333(3) |
| Si(1)－F(1)^1^ | 1.6699(16) | C(1)－C(1)^2^ | 1.471(5) |
| Si(1)－F(3) | 1.6606(17) | C(1)－C(4) | 1.371(4) |
| Si(1)－F(3)^1^ | 1.6605(17) | C(4)－C(3) | 1.387(4) |
| Si(1)－F(2) | 1.7025(19) | C(5)－C(3) | 1.371(4) |
| Si(1)－F(2)^1^ | 1.7026(19) | C(5)－C(2) | 1.368(4) |
| N(1)－C(1) | 1.352(3) |  |  |

| **Bond Angles (deg) for (C_6_H_5_N_2_)_2_SiF_6_** | | | |  |
| --- | --- | --- | --- | --- |
| F(1)－Si(1)－F(1)^1^ | 180.0 | F(3)－Si(1)－F(2)^1^ | 90.61(8) |  |
| F(1)^1^－Si(1)－F(2) | 89.42(7) | F(3)^1^－Si(1)－F(2)^1^ | 89.39(8) |  |
| F(1)－Si(1)－F(2) | 90.58(7) | F(2)－Si(1)－F(2)^1^ | 180.0 |  |
| F(1)^1^－Si(1)－F(2)^1^ | 90.58(7) | C(5)－N(1)－C(2) | 123.20(19) |  |
| F(1)－Si(1)－F(2)^1^ | 89.42(7) | C(4)－C(1)－C(6) | 120.9(2) |  |
| F(3)－Si(1)－F(1) | 91.33(7) | C(4)－C(1)－C(3) | 119.7(2) |  |
| F(3)－Si(1)－F(1)^1^ | 88.67(7) | C(6)－C(1)－C(3) | 119.47(19) |  |
| F(3)^1^－Si(1)－F(1)^1^ | 91.33(7) | C(2)－C(4)－C(1) | 118.5(2) |  |
| F(3)^1^－Si(1)－F(1) | 88.67(7) | C(5)－C(6)－C(1) | 180.0(2) |  |
| F(3)^1^－Si(1)－F(3) | 180.0 | N(2)－C(3)－C(1) | 118.0(3) |  |
| F(3)－Si(1)－F(2) | 89.39(8) | N(1)－C(2)－C(4) | 119.6(2) |  |
| F(3)^1^－Si(1)－F(2) | 90.61(8) | N(1)－C(5)－C(6) | 119.9(2) |  |
|  |  |  |  |  |
| **Bond Angles (deg) for (C_10_H_10_N_2_)SiF_6_** | | | | |
| F(1)^1^－Si(1)－F(1) | 180.00(13) | F(3)－Si(1)－F(2)^1^ | 89.63(10) |  |
| F(1)－Si(1)－F(2) | 89.22(10) | F(3)^1^－Si(1)－F(2)^1^ | 90.37(10) |  |
| F(1)^1^－Si(1)－F(2) | 90.78(9) | F(2)－Si(1)－F(2)^1^ | 180.0 |  |
| F(1)－Si(1)－F(2)^1^ | 90.78(10) | C(2)－N(1)－C(1) | 122.6(3) |  |
| F(1)^1^－Si(1)－F(2)^1^ | 89.22(10) | N(1)－C(1)－C(1)^2^ | 117.4(3) |  |
| F(3)－Si(1)－F(1)^1^ | 89.39(8) | N(1)－C(1)－C(4) | 118.8(3) |  |
| F(3)－Si(1)－F(1) | 90.61(8) | C(4)－C(1)－C(1)^2^ | 123.7(3) |  |
| F(3)^1^－Si(1)－F(1) | 89.39(8) | C(1)－C(4)－C(3) | 119.4(3) |  |
| F(3)^1^－Si(1)－F(1)^1^ | 90.61(8) | C(2)－C(5)－C(3) | 119.2(3) |  |
| F(3)^1^－Si(1)－F(3) | 180.0 | C(5)－C(3)－C(4) | 120.0(3) |  |
| F(3)－Si(1)－F(2) | 90.37(10) | N(1)－C(2)－C(5) | 120.0(3) |  |
| F(3)^1^－Si(1)－F(2) | 89.63(10) |  |  |  |

Symmetry transformations used to generate equivalent atoms: ^1^2-*x*,1-*y*,1-*z* for (C_6_H_5_N_2_)_2_SiF_6_ and ^1^3/2-*x*,3/2-*y*,1-*z*; ^2^1-*x*,+*y*,1/2-*z* for (C_10_H_10_N_2_)SiF_6_.

**Table S3**. Fractional Atomic Coordinates (× 10^4^) and Equivalent Isotropic Displacement Parameters (Å^2^ × 10^3^) for (C_6_H_5_N_2_)_2_SiF_6_ and (C_10_H_10_N_2_)SiF_6_. *U*_eq_ is defined as 1/3 of the trace of the orthogonalized U*_ij_* tensor.

| **(C_6_H_5_N_2_)_2_SiF_6_** | | | | | |
| --- | --- | --- | --- | --- | --- |
| **Atom** | **Wyckoff site** | ***x*** | ***y*** | ***z*** | ***U*_eq_^a^*/*Å^2^** |
| Si1 | 1*g* | 10000 | 5000 | 5000 | 27.8(3) |
| F1 | 2*i* | 9811(2) | 3587(2) | 6932.3(19) | 54.3(4) |
| F3 | 2*i* | 9509(3) | 3489(2) | 4068(2) | 59.0(5) |
| F2 | 2*i* | 7232(2) | 6313(3) | 6005.1(19) | 62.4(5) |
| N1 | 2*i* | 5365(3) | 7406(3) | 3531(3) | 41.8(5) |
| N2 | 2*i* | 2362(4) | 8786(4) | -1745(3) | 58.0(6) |
| C1 | 2*i* | 3860(3) | 8144(3) | 820(3) | 31.1(5) |
| C4 | 2*i* | 2647(4) | 9303(3) | 2415(3) | 35.9(5) |
| C6 | 2*i* | 5874(4) | 6628(3) | 577(3) | 38.5(5) |
| C3 | 2*i* | 3018(4) | 8505(3) | -622(3) | 37.9(5) |
| C2 | 2*i* | 3454(4) | 8899(4) | 3775(3) | 40.5(6) |
| C5 | 2*i* | 6585(4) | 6282(4) | 1993(3) | 43.9(6) |

| **(C_10_H_10_N_2_)SiF_6_** | | | | | |
| --- | --- | --- | --- | --- | --- |
| **Atom** | **Wyckoff site** | ***x*** | ***y*** | ***z*** | ***U*_eq_^a^*/*Å^2^** |
| Si1 | 4*d* | 7500 | 7500 | 5000 | 30.3(4) |
| F1 | 8*f* | 7535.4(13) | 6733(3) | 6318.0(18) | 49.7(6) |
| F3 | 8*f* | 8721.4(12) | 7204(3) | 6058.8(18) | 51.5(6) |
| F2 | 8*f* | 7320.9(15) | 5053(3) | 4440(2) | 65.0(7) |
| N1 | 8*f* | 5931.7(18) | 3292(3) | 4404(3) | 30.2(7) |
| C1 | 8*f* | 5045(2) | 2756(4) | 3134(3) | 26.3(7) |
| C4 | 8*f* | 4298(2) | 2164(4) | 3105(3) | 30.8(8) |
| C5 | 8*f* | 5386(2) | 2643(4) | 5644(3) | 35.4(8) |
| C3 | 8*f* | 4468(2) | 2138(4) | 4372(3) | 34.2(8) |
| C2 | 8*f* | 6116(2) | 3228(4) | 5637(3) | 34.7(8) |

Table S4. Summary of related fluoride birefringent crystals.

| **Compound** | **Space group** | **Birefringence** | **Reference** |
| --- | --- | --- | --- |
| (C_10_H_10_N_2_)SiF_6_ | *C*2/*c* | Exp.0.583@546nm | This work |
| (C_6_H_5_N_2_)_2_SiF_6_ | *P*$\bar{1}$ | Exp.0.505@546nm | This work |
| [C_3_N_6_H_7_]_2_[B_3_O_3_F_4_(OH)] | *P*$\bar{1}$ | Cal.0.44@546nm | [11] |
| (C_3_N_6_H_7_)BF_4_·H_2_O | *P*$\bar{1}$ | Exp.0.37@546nm | [12] |
| K_2_Sn_2_(C_2_O_4_)_2_F_2_·H_2_O | *P*2_1_/*c* | Exp.0.301@546nm | [13] |
| (C_2_H_4_N_4_)_4_ZnSiF_6_·H_2_O | *P*$\bar{1}$ | Exp.0.282@546nm | [14] |
| [C_10_H_8_NO_2_]_2_SiF_6_·H_2_O | *Pna*2_1_ | Exp.0.282@550nm | [15] |
| CdF(C_6_H_4_NO_2_)(H_2_O) | *P*2_1_ | Exp.0.253@546.1nm | [16] |
| Sn(IO_3_)_2_F_2_ | *P*2_1_ | Cal.0.234@1064nm | [17] |
| RbGaF_3_(IO_3_) | *Pnma* | Cal.0.174@1064nm | [17] |
| [GaF(H2O)][IO3F] | *Pca*2_1_ | Cal.0.166@532nm | [18] |
| (C_3_N_6_H_7_)_2_SiF_6_·H_2_O | *P*2_1_/*n* | Cal.0.152@550nm | [19] |
| Na_3_SiS_3_F | *Cmc*2_1_ | Cal.0.15@1064nm | [20] |
| Pb_2_(SeO_3_)(SiF_6_) | *Pnma* | Exp.0.147@546nm | [21] |
| KBa_3_Zr_2_F_14_Cl | *P*$\bar{4}$/*m*2 | Cal.0.14@546nm | [22] |
| (C_2_H_7_N_4_O)_2_SiF_6_ | *P*$\bar{1}$ | Exp.0.135@546nm | [14] |
| β-Ba_2_[GaF_4_(IO_3_)_2_](IO_3_) | *P*2_1_ | Cal.0.135@1064nm | [23] |
| KBa_3_Hf_2_F_14_Cl | *P*$\bar{4}$/*m*2 | Cal.0.124@546nm | [22] |
| α-Ba_2_[GaF_4_(IO_3_)2](IO_3_) | *Pna*2_1_ | Cal.0.126@1064nm | [23] |
| K_3_Ba_2_Zr_6_F_31_ | *P*6_3_*mc* | Cal.0.08@1064nm | [24] |
| Ba2Ga2F6(IO3)(PO4) | *P*2_1_/*c* | Exp.0.072@550nm | [25] |
| Ba2[GaF5(IO3F)] | *P*2_1_/*c* | Exp.0.068@550nm | [26] |
| K_3_Na(TaF_7_)(SiF_6_) | *Immm* | Cal.0.045@253.7nm | [27] |
| MgF_2_ | *P*4_2_/*mnm* | Exp.0.012@546nm | [28] |
| Li_2_KAl_2_F_9_ | *C*2/*m* | Cal.0.0056@1064nm | [29] |
| BaAlF5 | *I*4/*m* | Cal.0.0044@1064nm | [29] |
| Na_2_SiF_6_ | *P*321 | Cal.0.002@200-1200nm | [30] |

Table S5. The assignment of the IR absorption peaks for (C_6_H_5_N_2_)_2_SiF_6_ and (C_10_H_10_N_2_)SiF_6_.

| **Assignment (cm^-1^)** | **(C_6_H_5_N_2_)_2_SiF_6_** |
| --- | --- |
| **V(N-H)** | **3260,3108,3066** |
| **V(C-H)** | **2932-2696,1643-1562,812-636** |
| **V(C≡N)** | **2221** |
| **V(C-C)** | **1442-1296** |
| **V(C-N)** | **1216-939** |
| **V(Si-F)** | **520** |

| **Assignment (cm^-1^)** | **(C_10_H_10_N_2_)SiF_6_** |
| --- | --- |
| **V(N-H)** | **3089** |
| **V(C-H)** | **2777,1630-1529,789-596** |
| **V(C-C)** | **1441-1286** |
| **V(C-N)** | **1251-976** |
| **V(Si-F)** | **533,468** |

Table S6. Hydrogen atom coordinates (Å × 10^4^) and isotropic displacement parameters (Å^2^ × 10^3^) for (C_6_H_5_N_2_)_2_SiF_6_ and (C_10_H_10_N_2_)SiF_6_.

| **(C_6_H_5_N_2_)_2_SiF_6_** | | | | | **(C_10_H_10_N_2_)SiF_6_** | | | | |
| --- | --- | --- | --- | --- | --- | --- | --- | --- | --- |
| **Atom** | ***x*** | ***y*** | ***z*** | **U(eq)** | **Atom** | ***x*** | ***y*** | ***z*** | **U(eq)** |
| H1 | 5833 | 7155 | 4410 | 50 | H1 | 6395 | 3689 | 4417 | 36 |
| H4 | 1312 | 10335 | 2565 | 43 | H4 | 3682 | 1783 | 2244 | 37 |
| H6 | 6715 | 5870 | -514 | 46 | H5 | 5511 | 2588 | 6501 | 42 |
| H2 | 2673 | 9663 | 4861 | 49 | H3 | 3960 | 1779 | 4359 | 41 |
| H5 | 7917 | 5265 | 1880 | 53 | H2 | 6741 | 3582 | 6492 | 42 |

Table S7. Hydrogen bonds for (C_6_H_5_N_2_)_2_SiF_6_ and (C_10_H_10_N_2_)SiF_6_.

| **compounds** | **D** | **H** | **A** | **d(D-H)/Å** | **d(H-A)/Å** | **d(D-A)/Å** | **D-H-A/°** |
| --- | --- | --- | --- | --- | --- | --- | --- |
| **(C_6_H_5_N_2_)_2_SiF_6_** | N1 | H1 | F2 | 0.86 | 1.82 | 2.675(2) | 170.5 |
| **(C_10_H_10_N_2_)SiF_6_** | N1 | H1 | F2 | 0.86 | 1.87 | 2.719(3) | 169.0 |

Table S8. Functional unit densities of (C_6_H_5_N_2_)_2_SiF_6_ and (C_10_H_10_N_2_)SiF_6_.

| **compounds** | **Density of organic**  **groups (n V^−1^) (Å^−3^)** | **Density of [SiF_6_]^2−^**  **(n V^−1^) (Å^−3^)** |
| --- | --- | --- |
| **(C_6_H_5_N_2_)_2_SiF_6_** | 5.79 × 10^-3^ | 2.89 × 10^-3^ |
| **(C_10_H_10_N_2_)SiF_6_** | 3.64 × 10^-3^ | 3.64 × 10^-3^ |

**
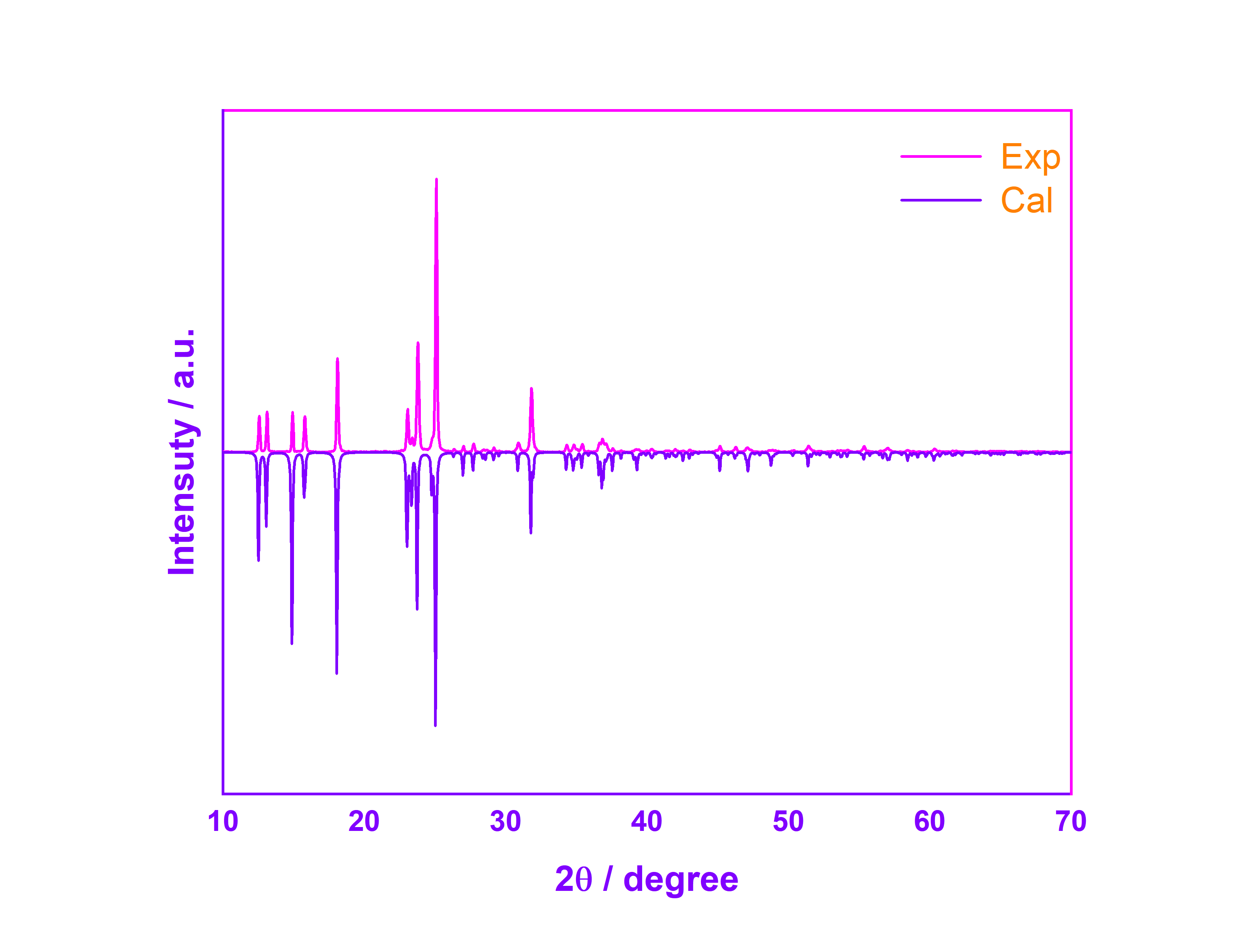

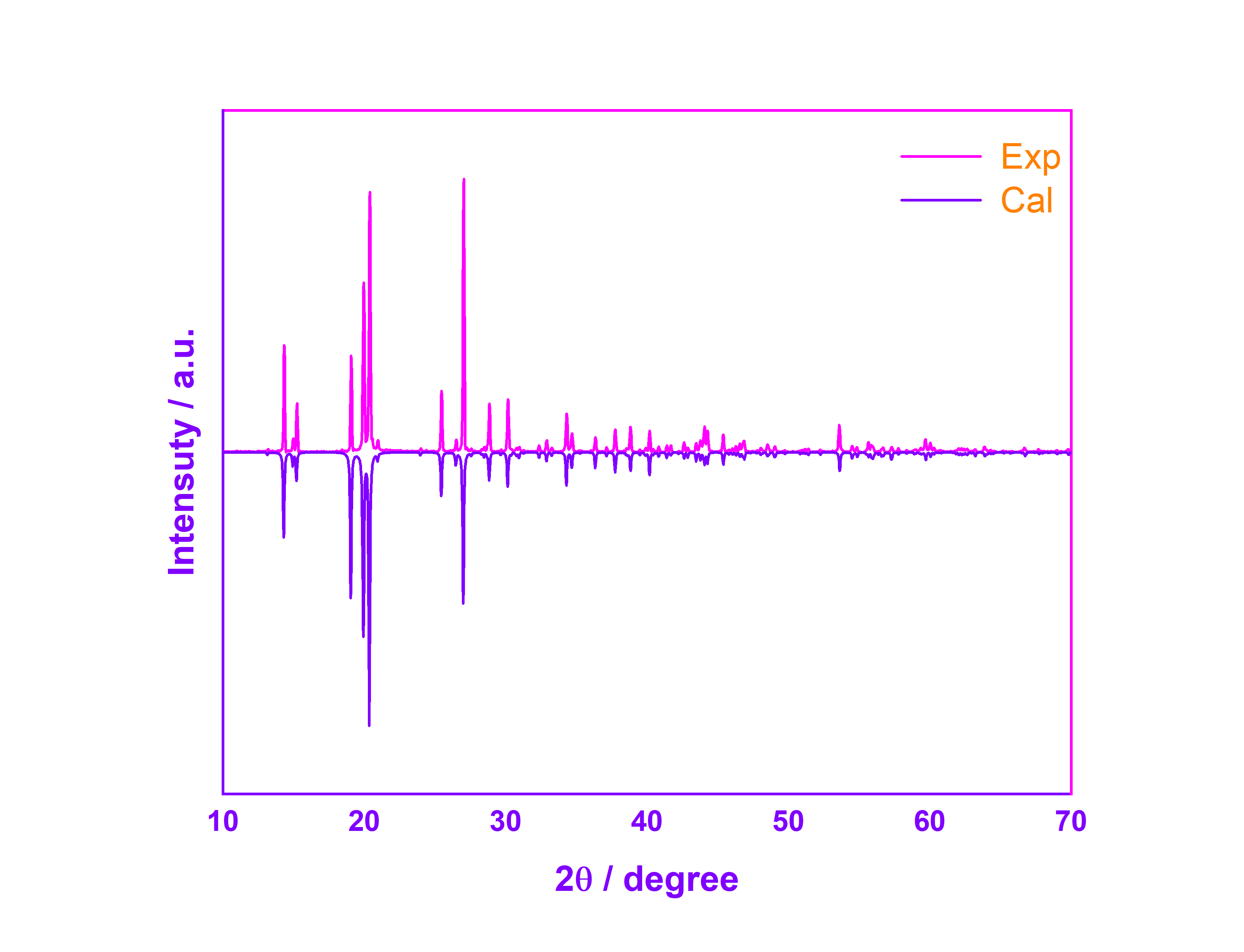
**

**(a) (b)**

**Figure S1.** Powder X-ray diffraction patterns of (a) (C_6_H_5_N_2_)_2_SiF_6_ and (b) (C_10_H_10_N_2_)SiF_6_.

**
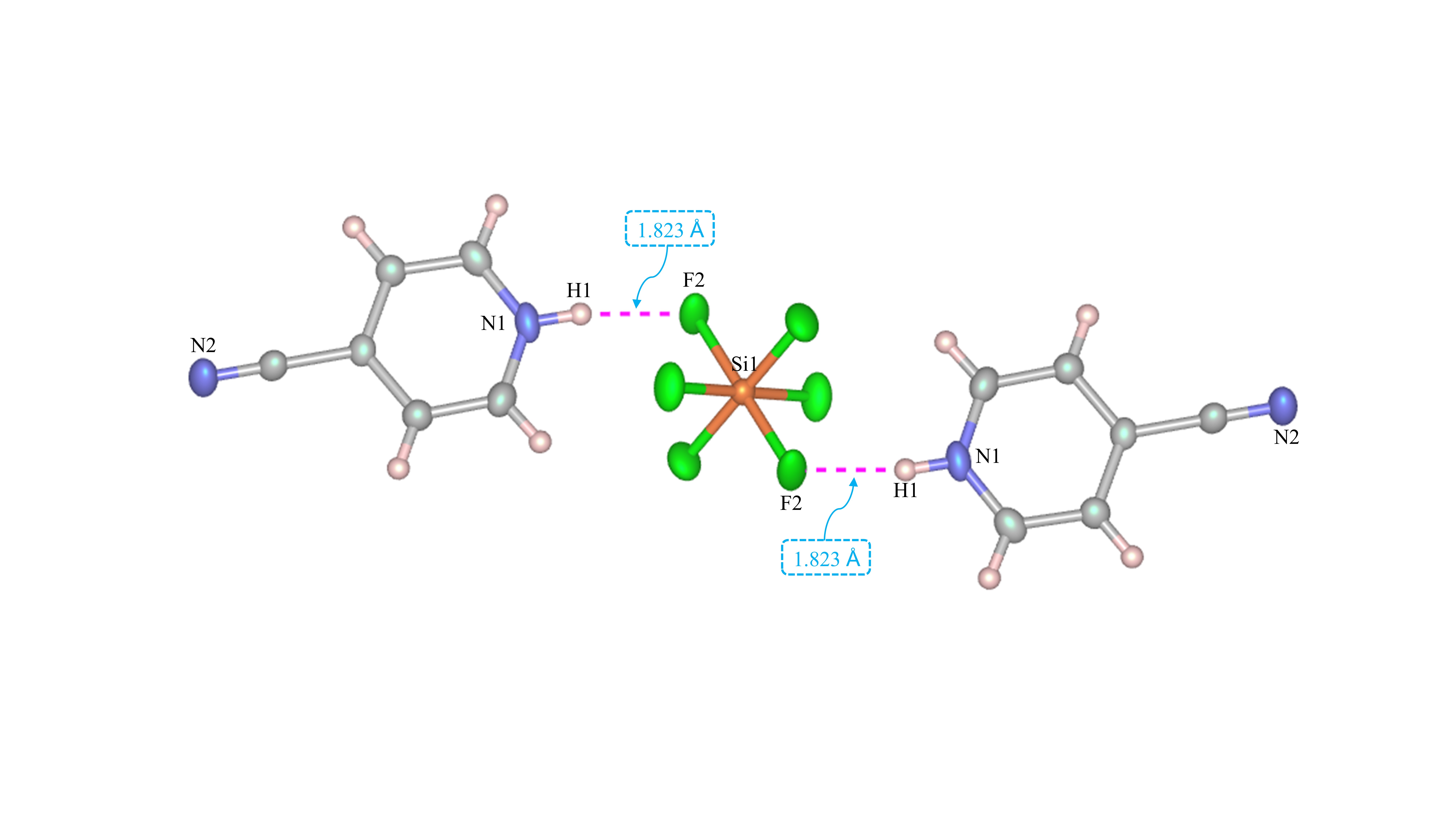
**

**(a)**

**
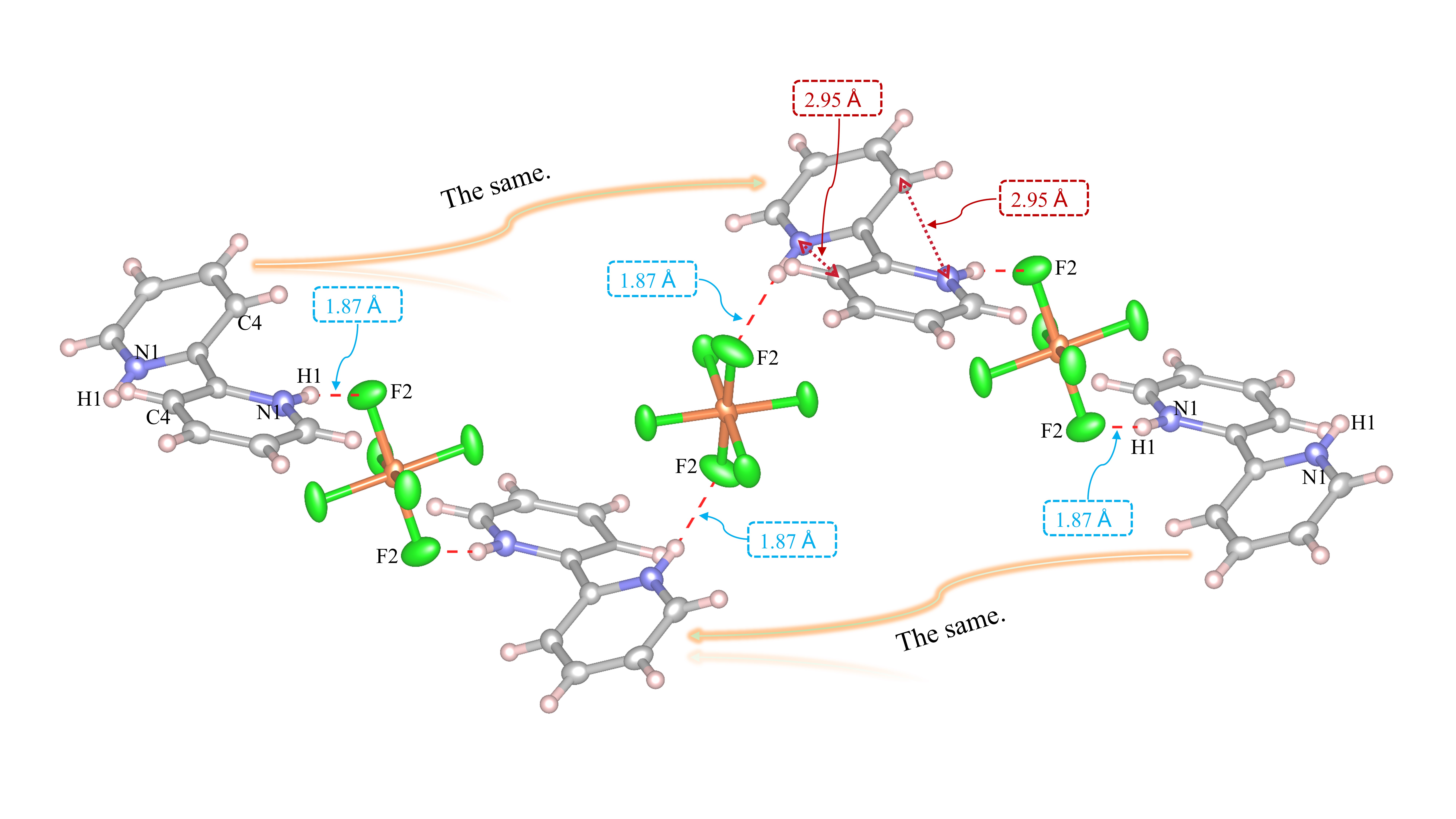
**

**(b)**

**Figure S2.** Hydrogen connections in (a) (C_6_H_5_N_2_)_2_SiF_6_ and (b) (C_10_H_10_N_2_)SiF_6_.

**
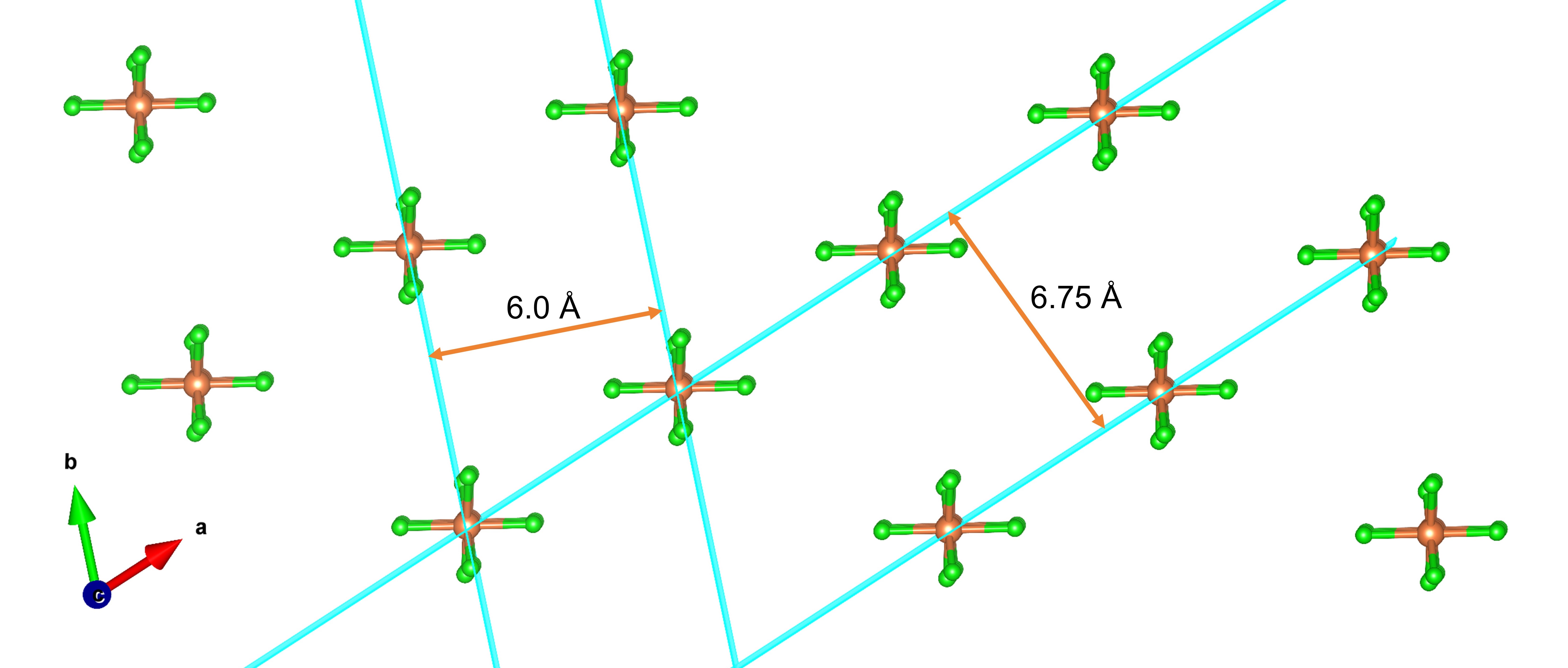
**

**(a)**

**
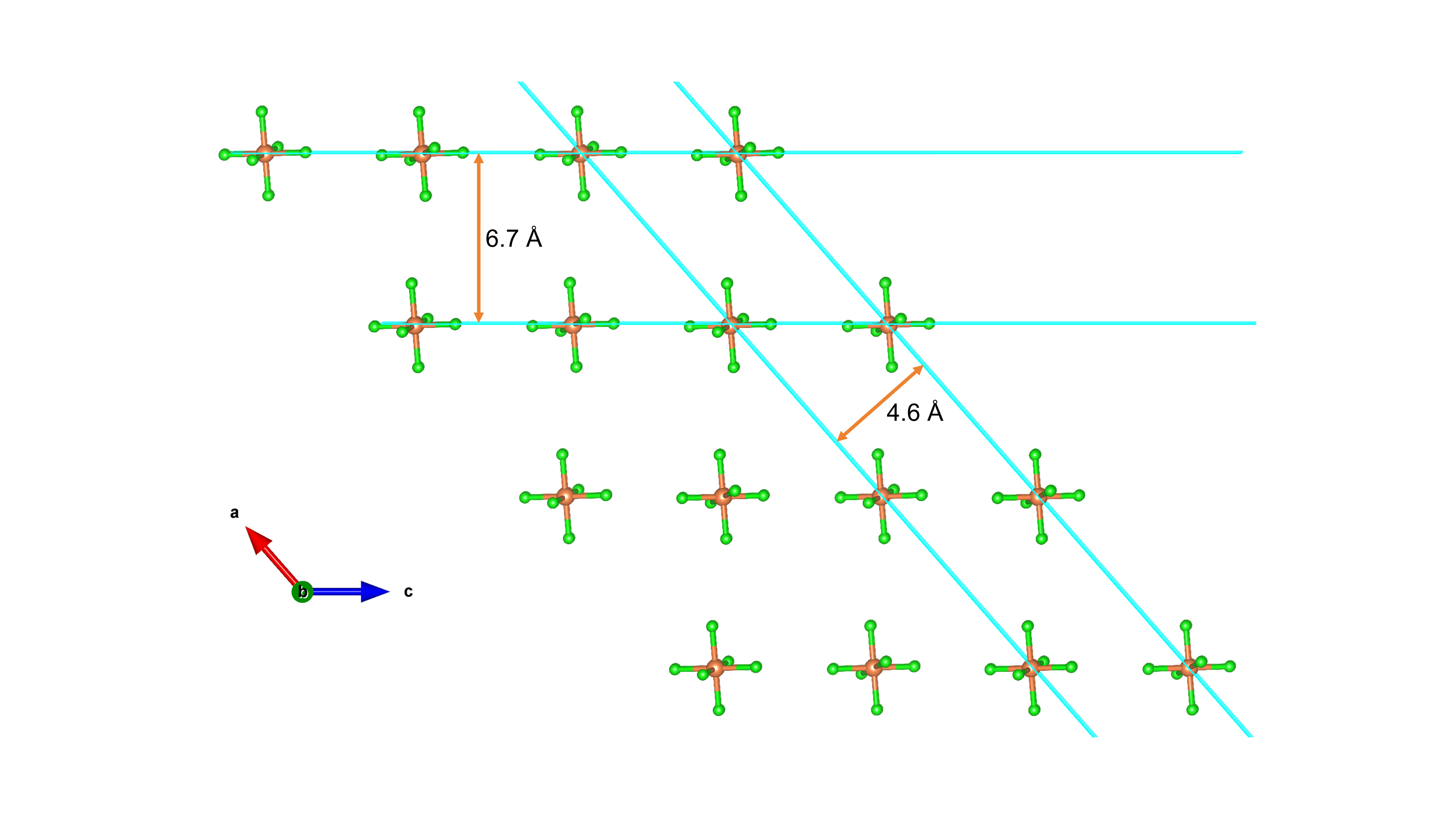
**

**(b)**

**Figure S3.** The parallel arrangements and planar spacing of the [SiF_6_]^2-^ anion groups in (a) (C_6_H_5_N_2_)_2_SiF_6_ and (b) (C_10_H_10_N_2_)SiF_6_.

**(a) (b)**

**Figure S4.** EDS images of (a) (C_6_H_5_N_2_)_2_SiF_6_ and (b) (C_10_H_10_N_2_)SiF_6_.


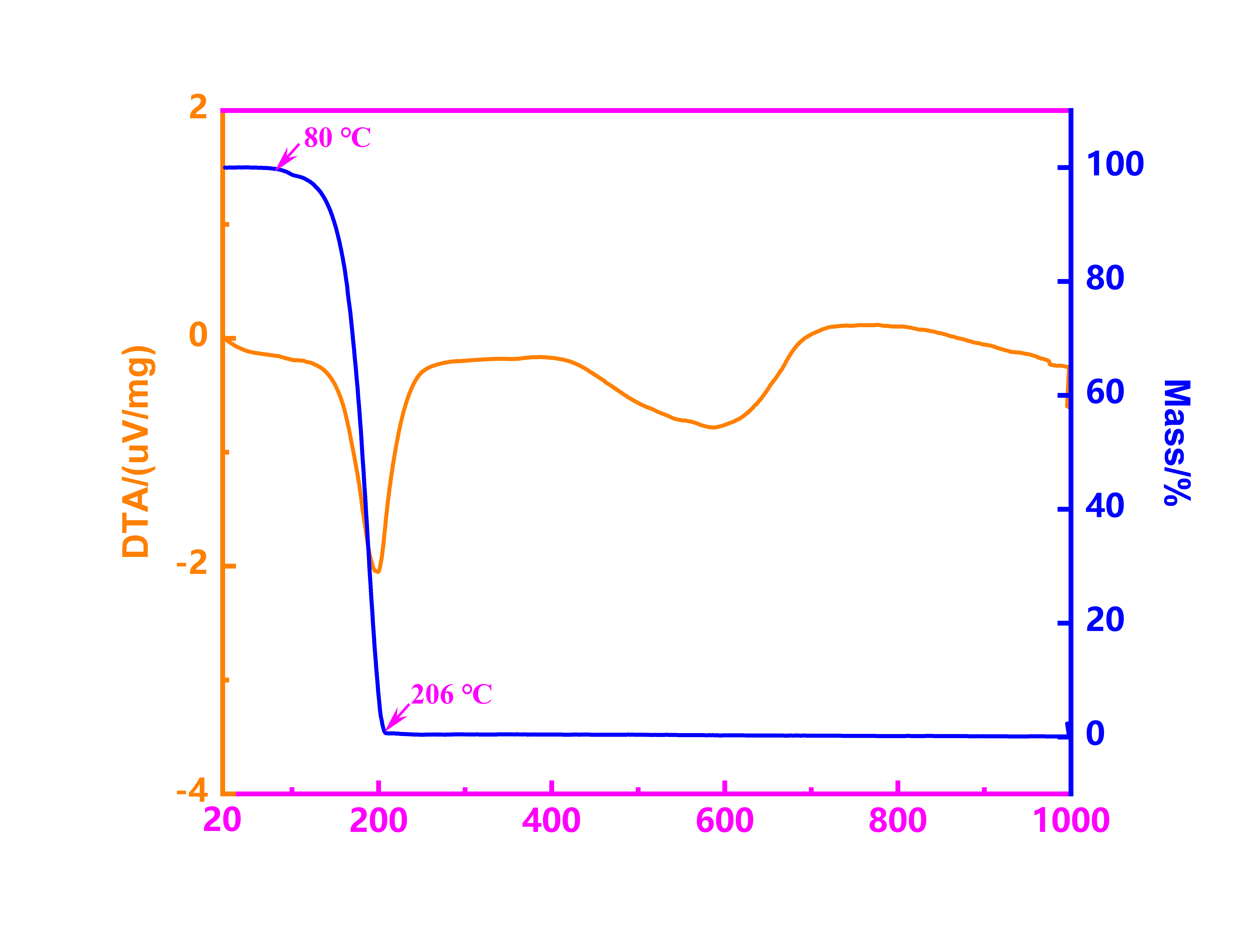

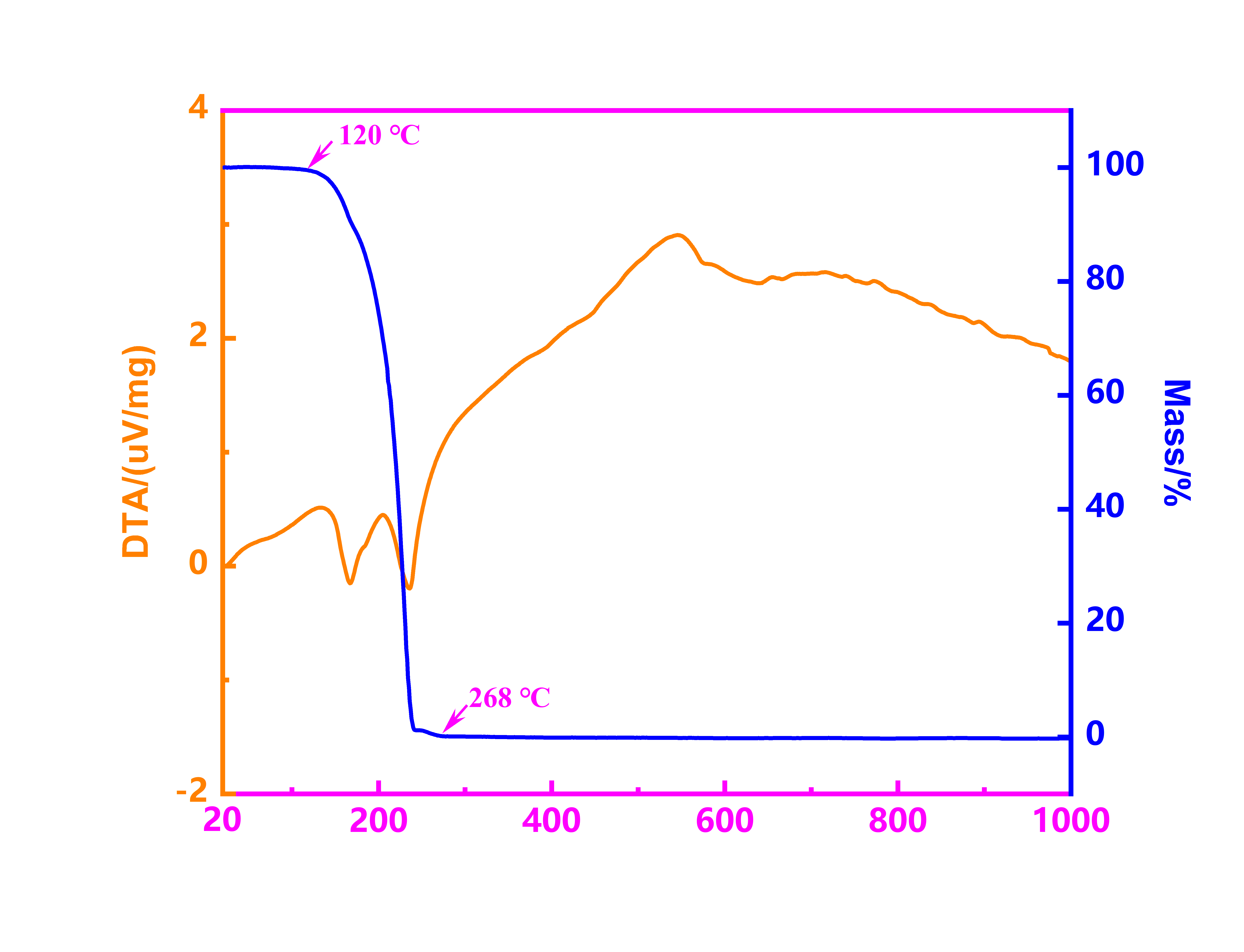


**(a) (b)**

**Figure S5.** TG-DTA curves of (a) (C_6_H_5_N_2_)_2_SiF_6_ and (b) (C_10_H_10_N_2_)SiF_6_.


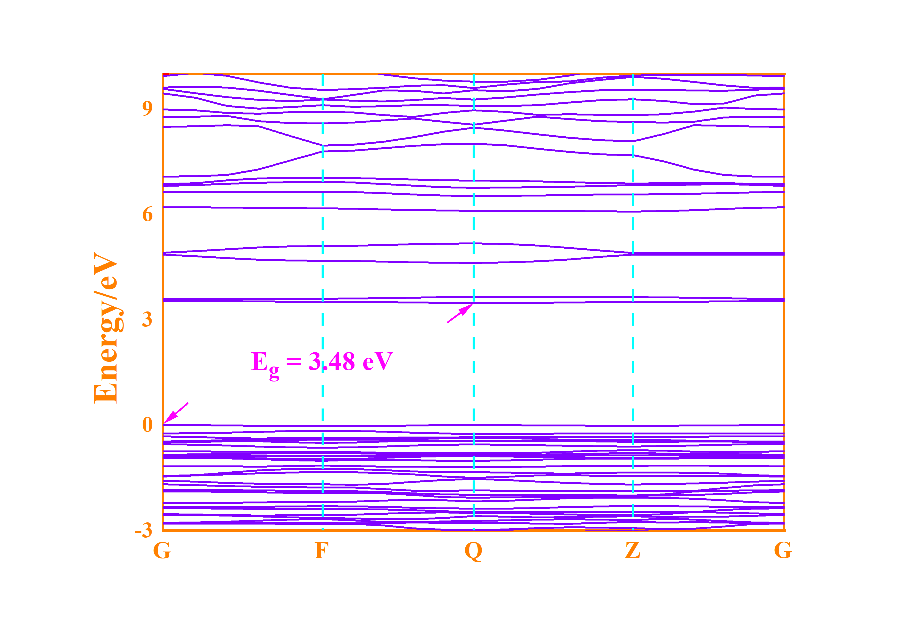

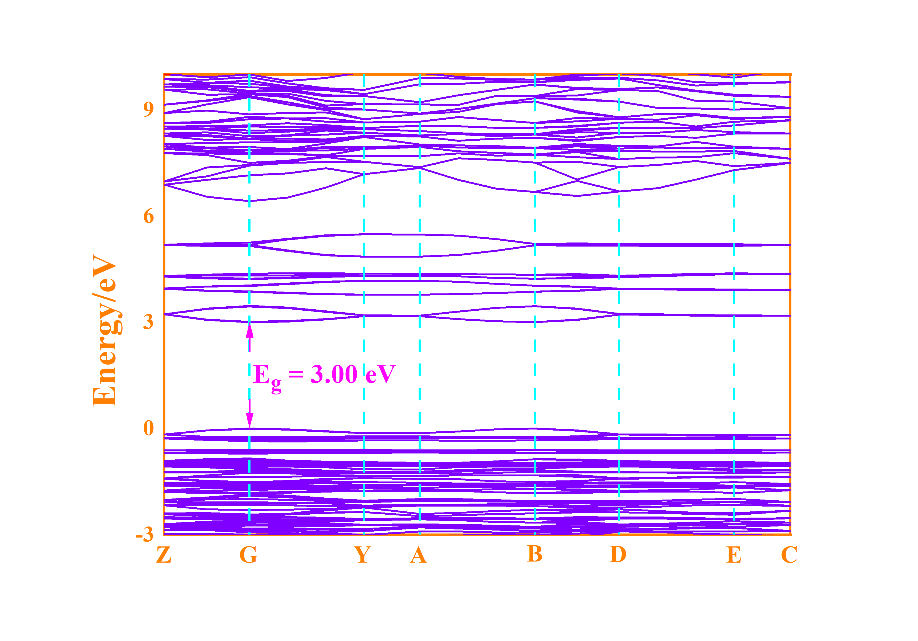


**(a)** **(b)**

**Figure S6.** Calculated band structures of (a) (C_6_H_5_N_2_)_2_SiF_6_ and (b) (C_10_H_10_N_2_)SiF_6_.


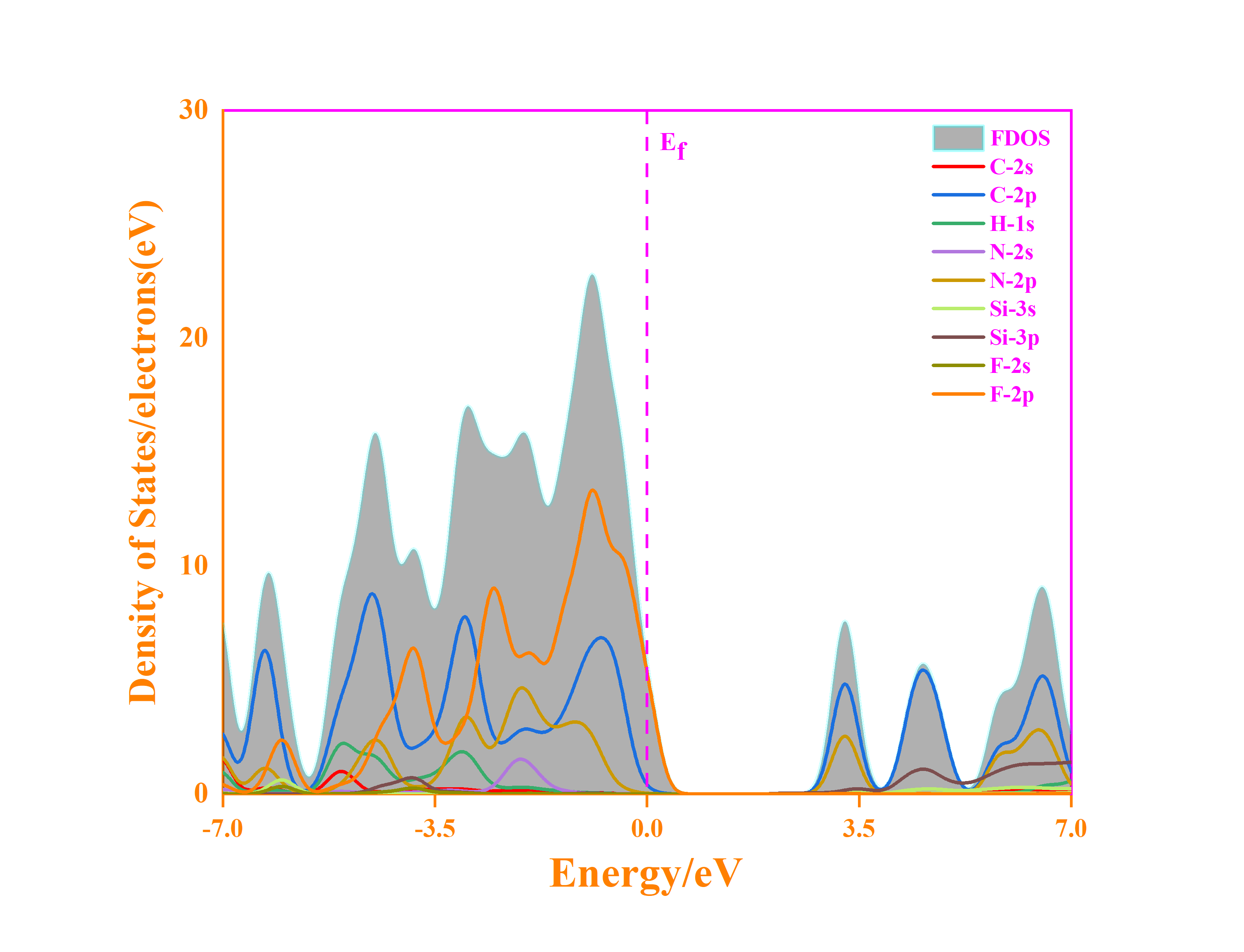
 **
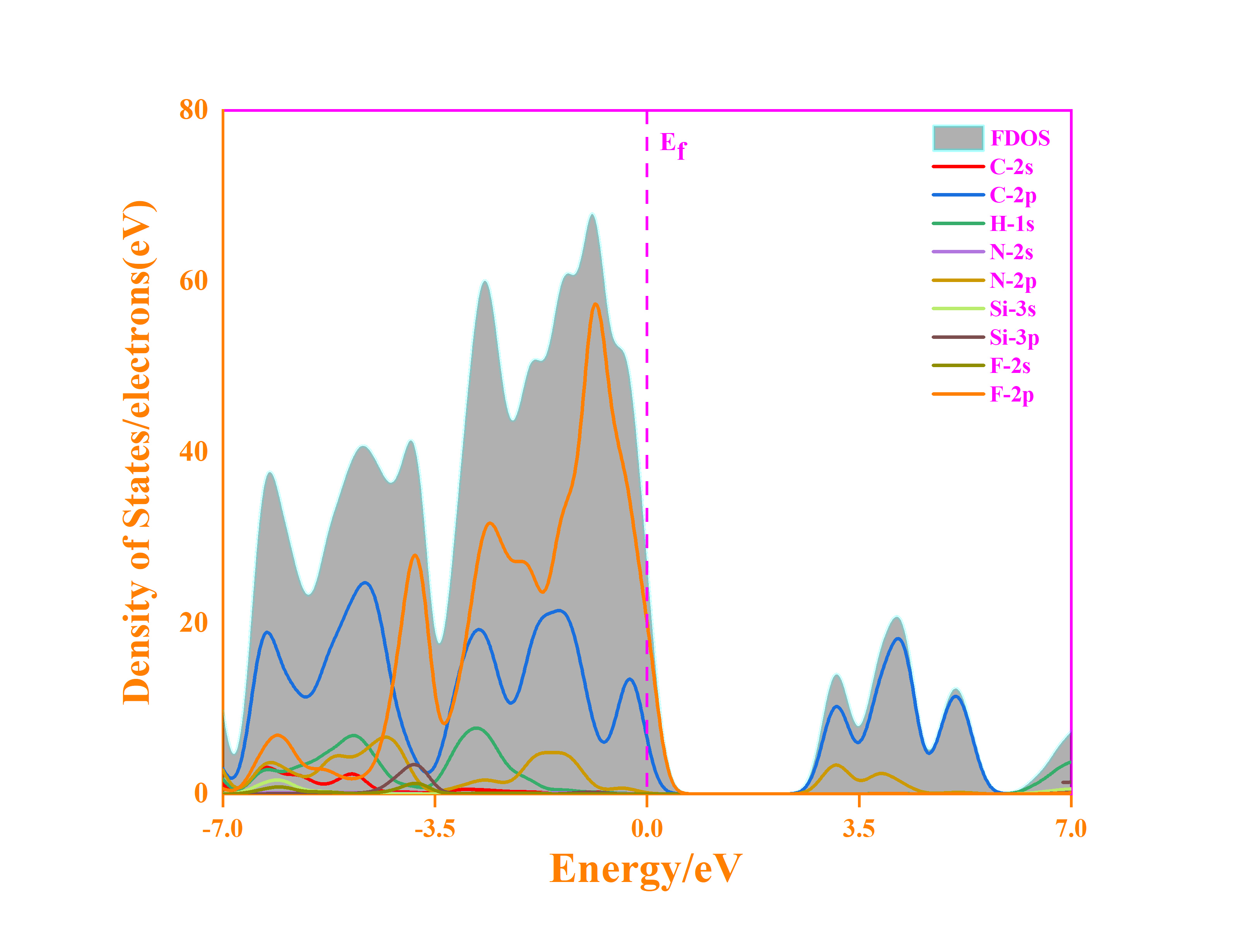
**

**(a) (b)**

**Figure S7.** Density of states (DOS) of (a) (C_6_H_5_N_2_)_2_SiF_6_ and (b) (C_10_H_10_N_2_)SiF_6_. The fermi level is set at 0 eV.

**
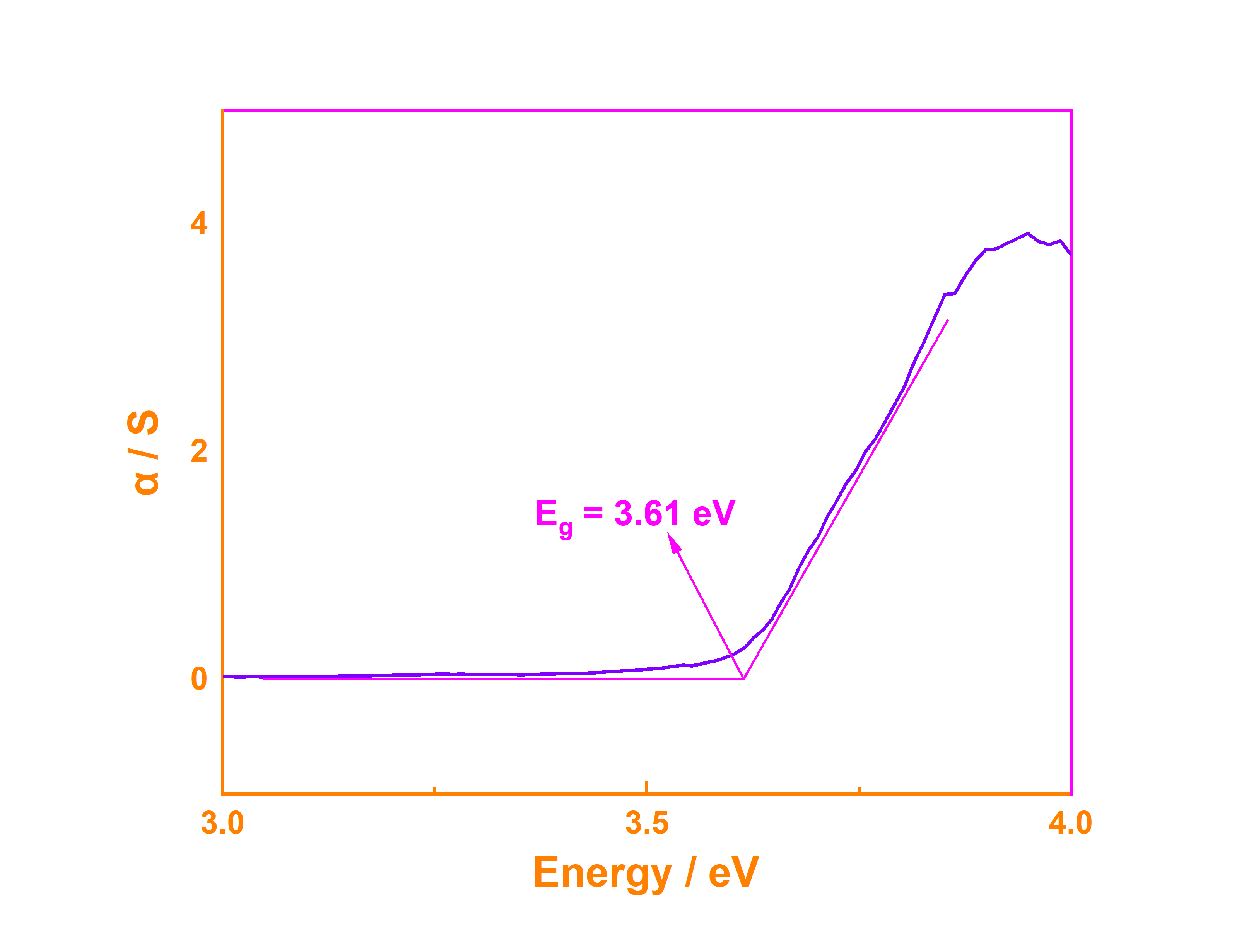

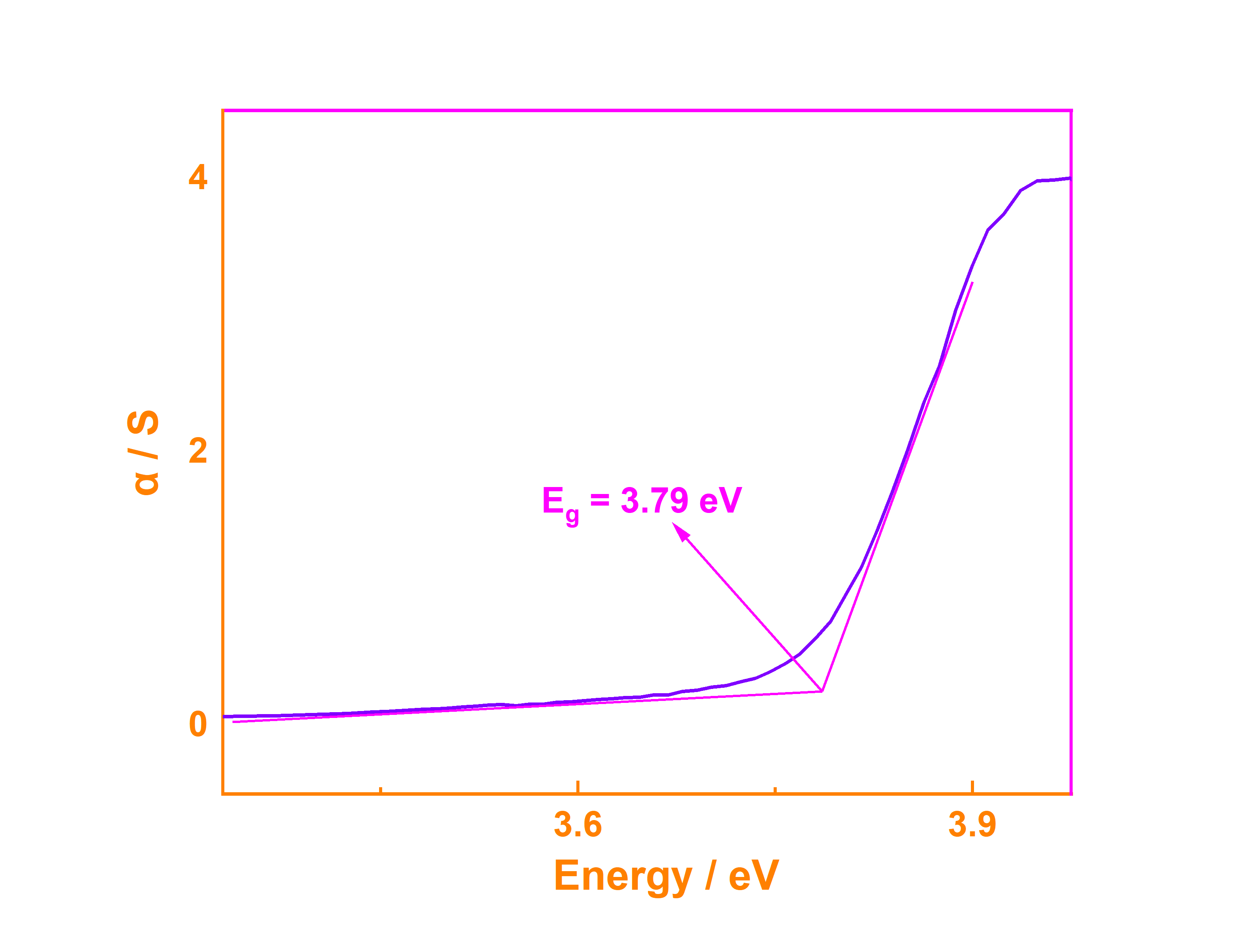

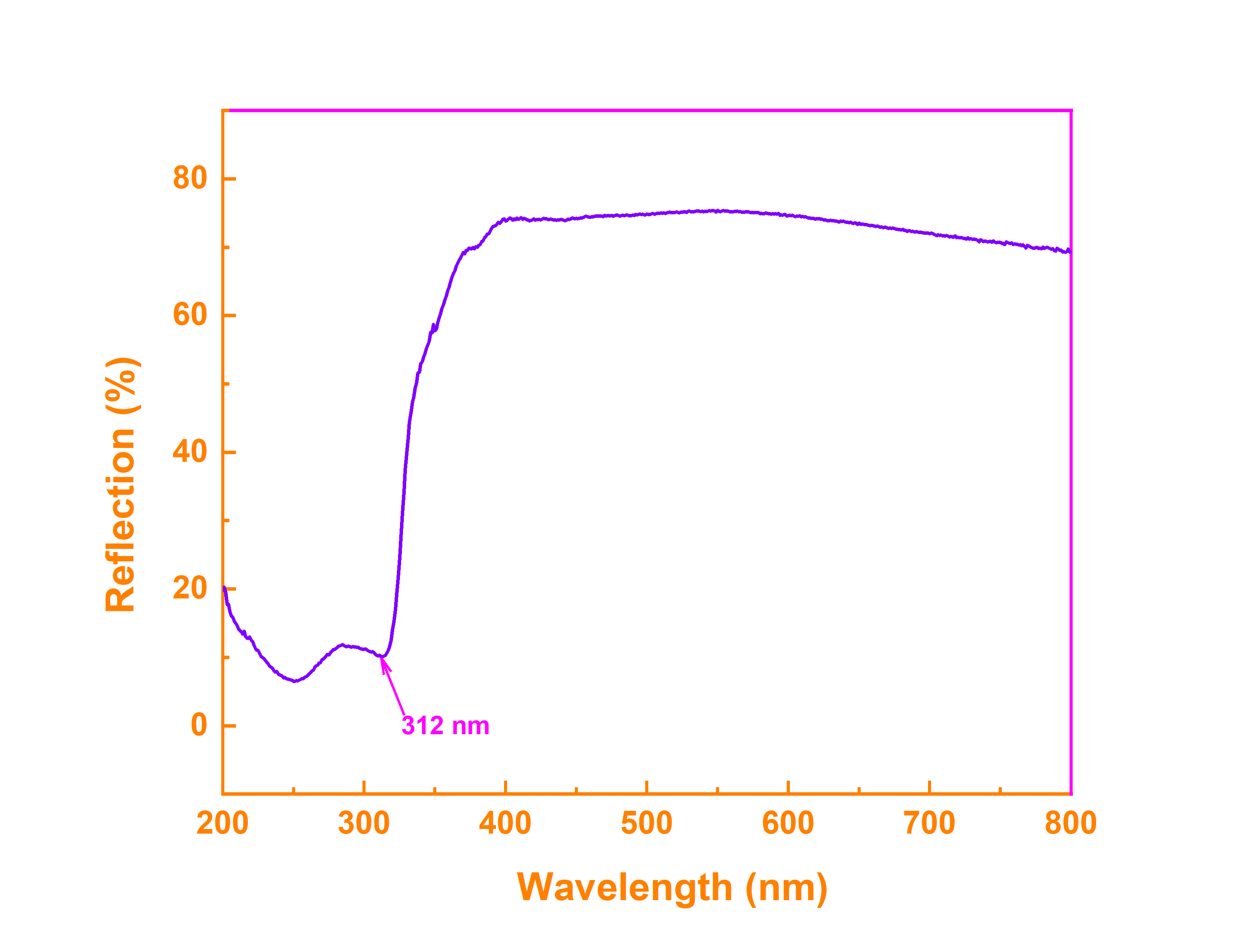

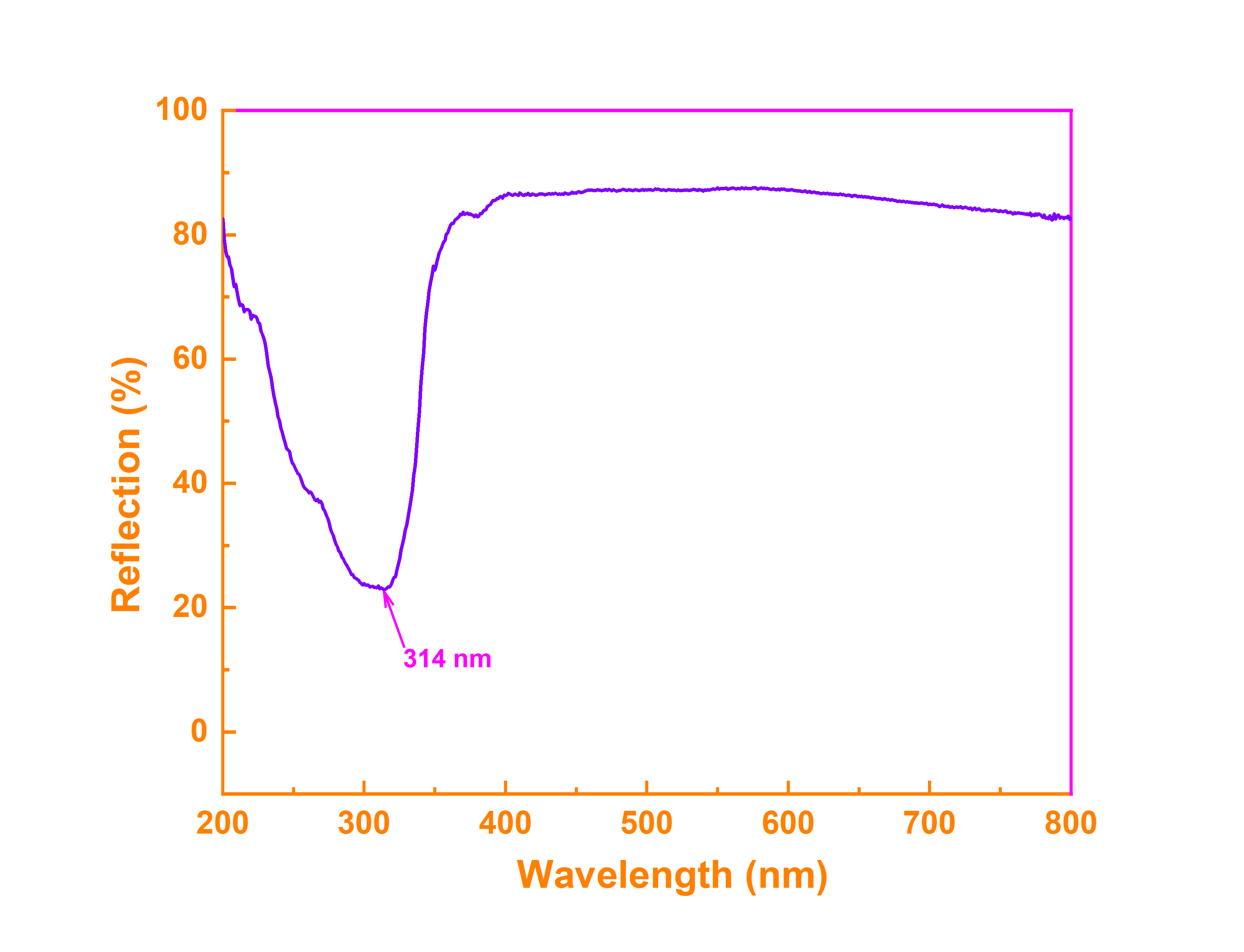
**

**(a) (b)**

**Figure S8.** The UV-vis diffuse reflectance spectra and optical bandgaps of (a) (C_6_H_5_N_2_)_2_SiF_6_ and (b) (C_10_H_10_N_2_)SiF_6._


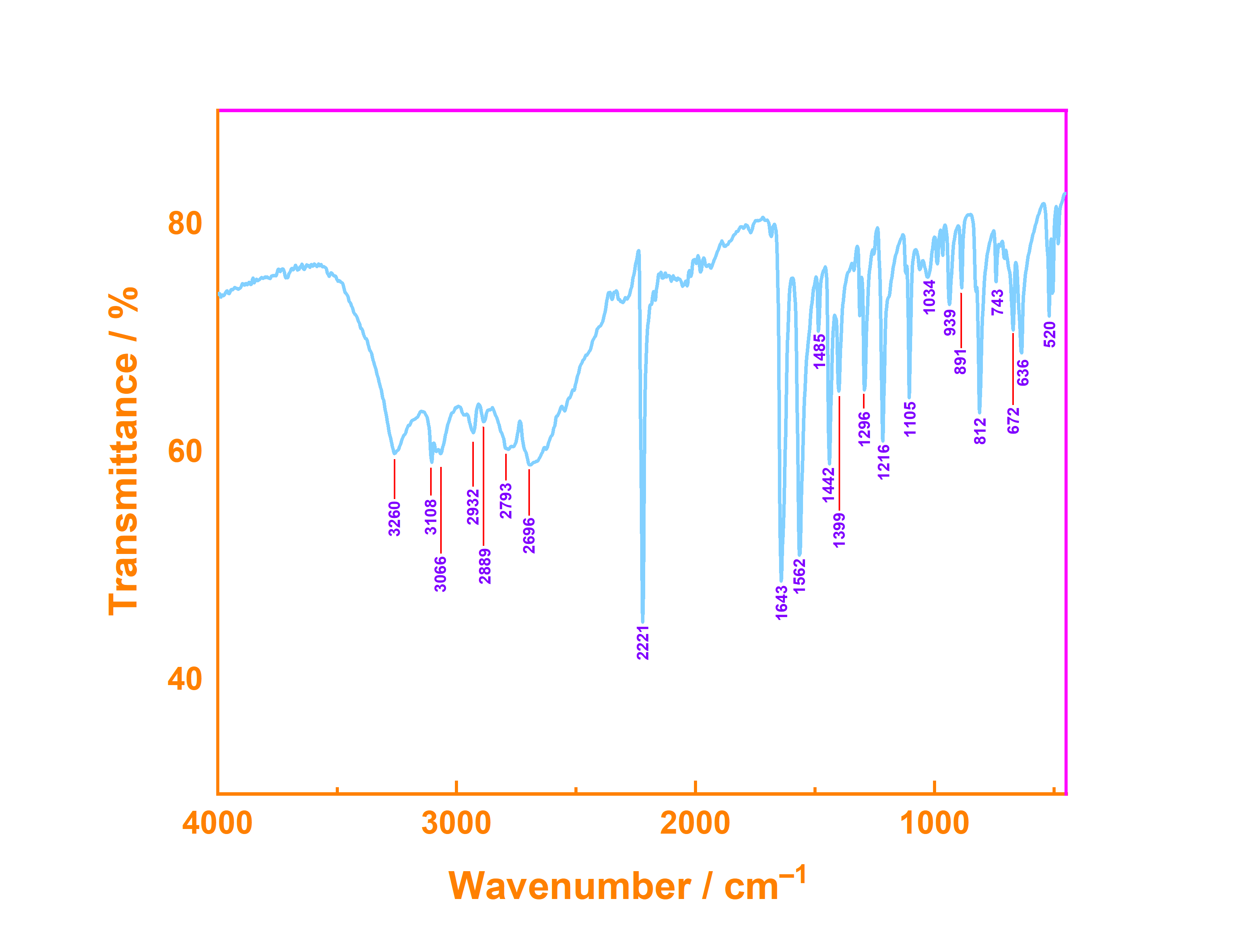

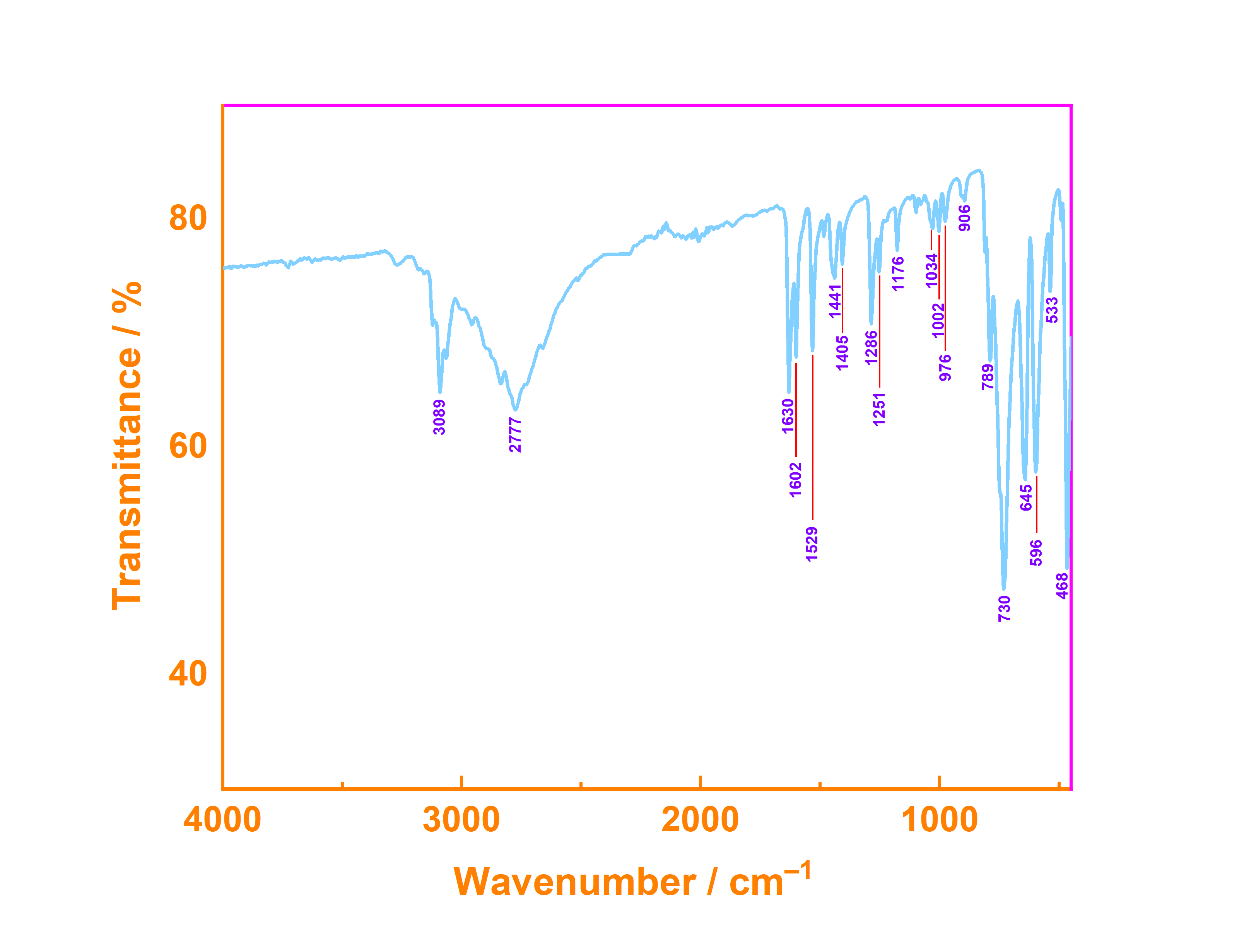


**(a) (b)**

**Figure S9.** IR spectra of (a) (C_6_H_5_N_2_)_2_SiF_6_ and (b) (C_10_H_10_N_2_)SiF_6_.


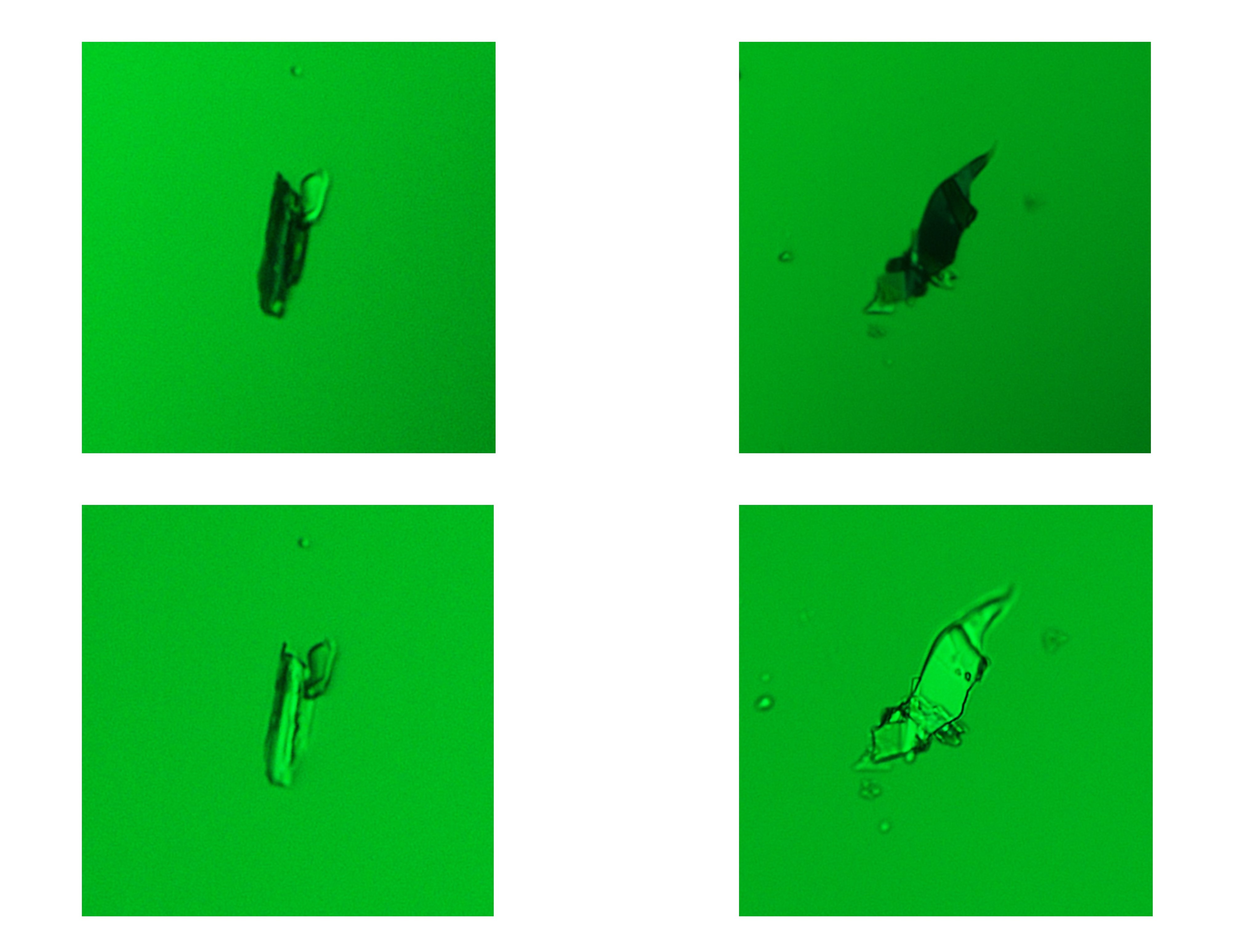


**(a) (b)**

**Figure S10.** Crystal photos of (a) (C_6_H_5_N_2_)_2_SiF_6_ and (b) (C_10_H_10_N_2_)SiF_6_ in their initial state and crystal photos after complete extinction.


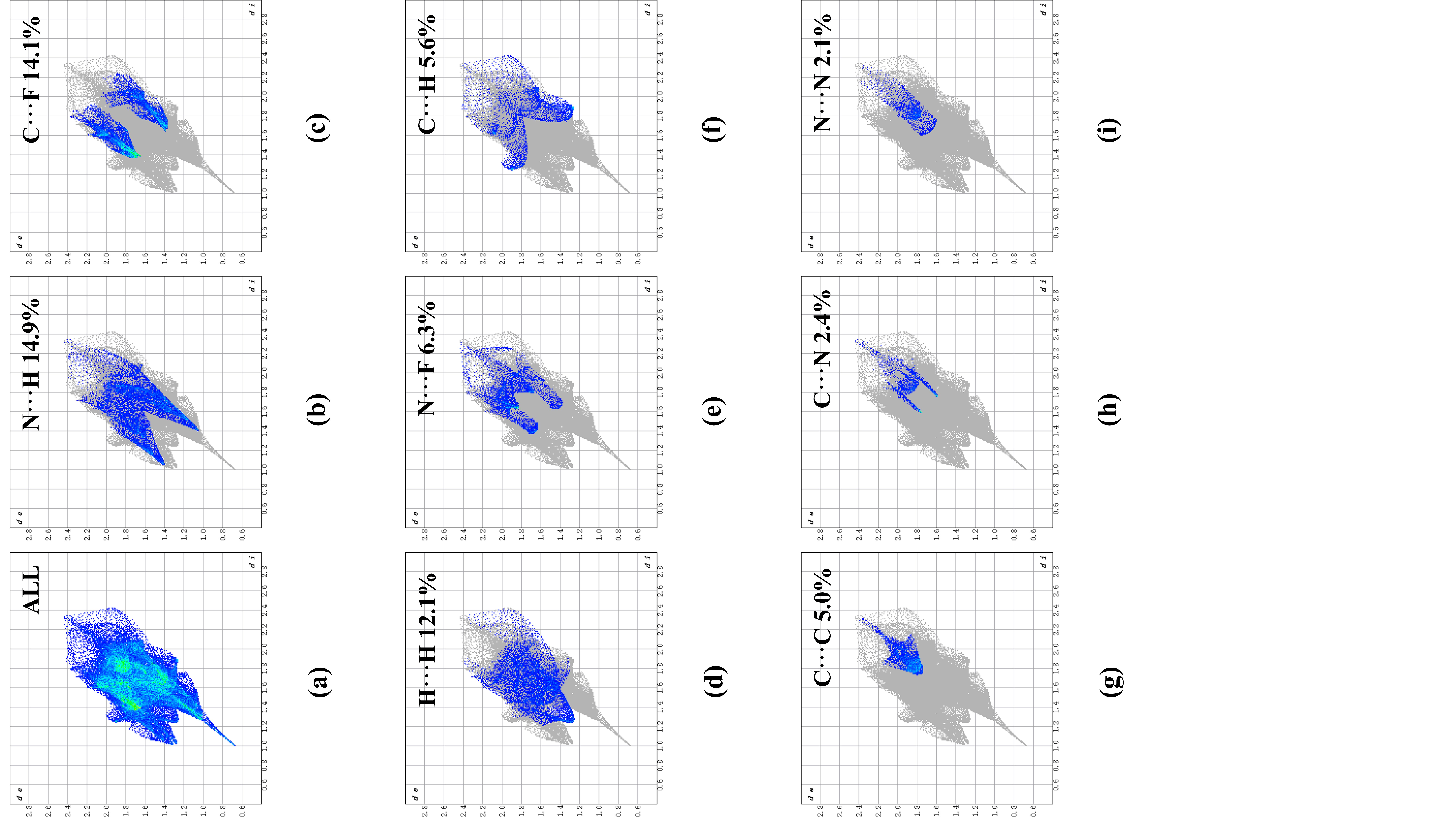


**Figure S11.** 2D fingerprint plots for (a) overall interactions and (b−i) the rest individual interactions of atom types in crystal packing of (C_6_H_5_N_2_)_2_SiF_6_. Here, *d*_e_ and *d*_i_ represent distances from the Hirshfeld surfaces to the nearest nucleus outside and inside the surface, respectively.


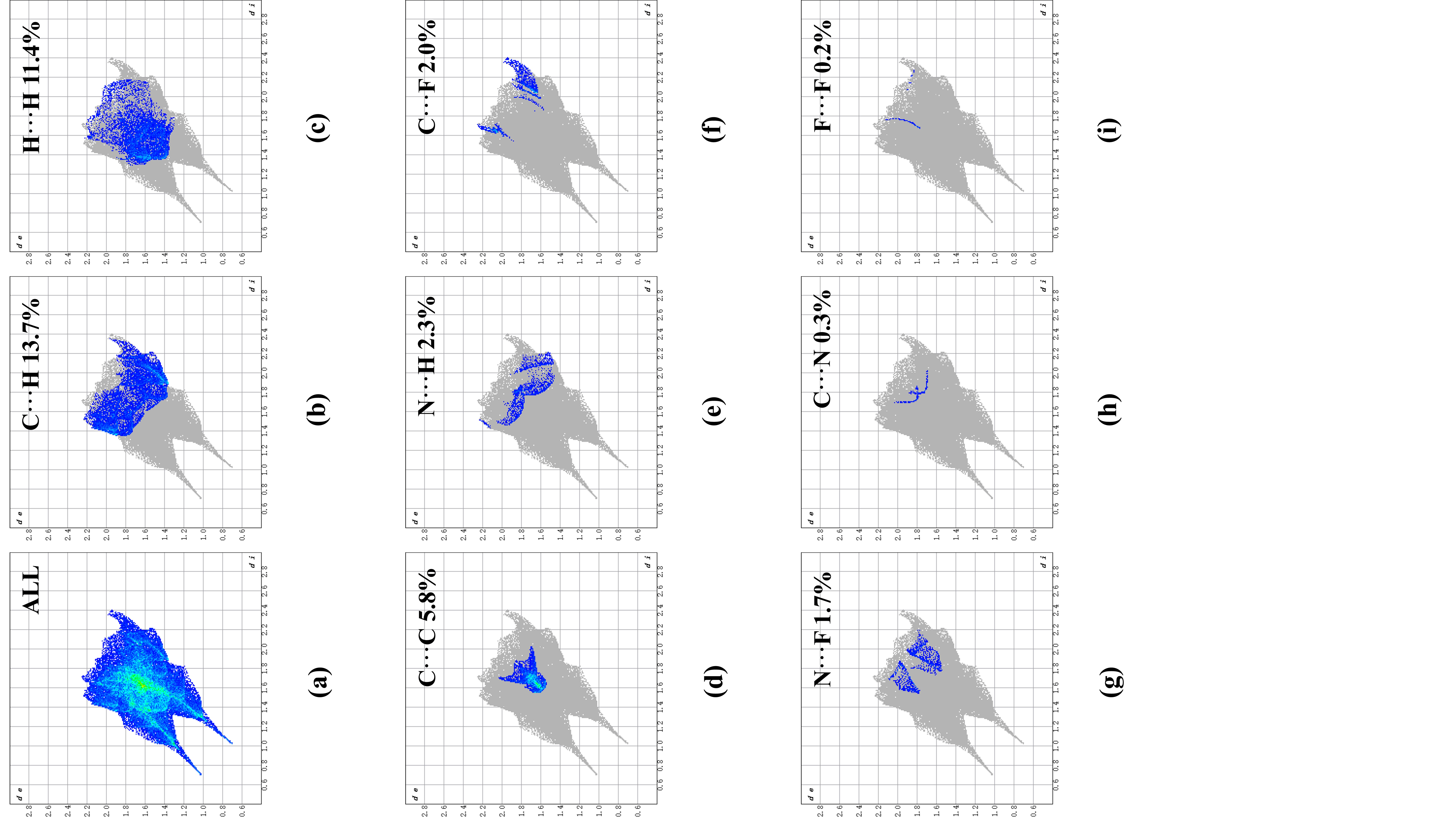


**Figure S12.** 2D fingerprint plots for (a) overall interactions and (b−i) the rest individual interactions of atom types in crystal packing of (C_10_H_10_N_2_)SiF_6_. Here, *d*_e_ and *d*_i_ represent distances from the Hirshfeld surfaces to the nearest nucleus outside and inside the surface, respectively.


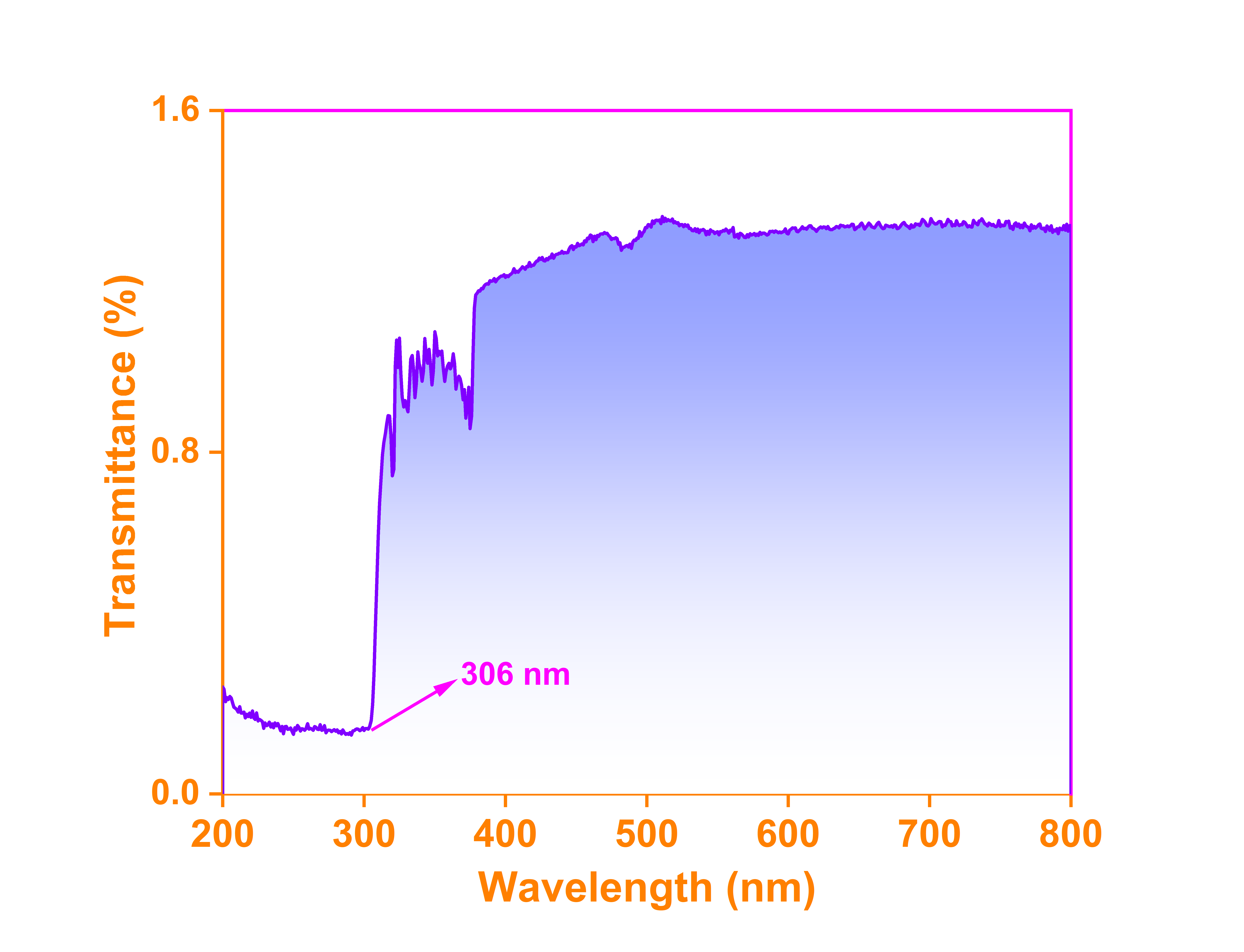


**Figure S13.** UV–Vis–NIR transmission spectrum of (C_6_H_5_N_2_)_2_SiF_6_.

**References:**

1. O. V. Dolomanov, L. J. Bourhis, R. J. Gildea, J. A. K. Howard and H. Puschmann, ICMCB UMR CNRS 5026, *J. Appl. Crystallogr*., **2009**, 42, 339.
2. G. Sheldrick, Crystal structure solution with ShelXT, *Acta Crystallogr*., *Sect. C: Struct. Chem*., **2015**, 71, 3.
3. H. Zhou, M. Cheng, D. D. Chu, X. Liu, R. An, S. L. Pan, Z. H. Yang, *Angew. Chem. Int. Ed*. **2025**, 64, e202413680.
4. J. Chen, M. B. Xu, H. Y. Wu, J. Y. Wu, K. Z. Du, *Angew. Chem. Int. Ed*. **2024**, 63, e202411503.
5. Segall M D, Lindan P J D, Probert M J, et al. First-principles simulation: ideas, illustrations and the CASTEP code. *J. Phys. Condens. Matter*, **2002**, 14, 2717.
6. J. P. Perdew, K. Burke, M. Ernzerhof, Generalized gradient approximation made simple. *Phys. Rev. Lett*., **1996**, 77, 3865-3868.
7. K. Kobayashi. Norm-conserving pseudopotential database (NCPS97). *Computational Materials Science*, **1999**, 14, 72-76.
8. Vanderbilt D. Soft self-consistent pseudopotentials in a generalized eigenvalue formalism. *Physical Review B*, **1990**, 41, 7892.
9. J. P. Perdew and M. Levy, Physical Content of the Exact Kohn Sham Orbital Energies: Band Gaps and Derivative Discontinuities, *Phys. Rev. Lett*., **1983**, 51, 1884-1887.
10. R. P. J. Cooney and J. R. Hall, Raman spectra of crystalline hydrolysis products of aqueous solutions of mercury(II) nitrate, *Aust. J. Chem*., **1972**, 25, 1159-1168.
11. C. Y. Jin, F. M. Li, Z. H. Yang, S. L. Pan, & M. Mutailipu, *J. Mater. Chem. C*, **2022**,10, 6590-6595.
12. H. W. Jia, D. Xu, Z. J. Li, M. Arif, Y. S. Jiang, X. L. Hou, *Inorg. Chem. Front*. **2024**,11, 8331-8338.
13. L. L. Ren, L. S. Cheng, X. N. Zhou, J. X. Ren, L. L. Cao, L. Huang, X. H. Dong, Y. Q. Zhou, D. J. Gao, G. H. Zou, *Inorg. Chem. Front*. **2023**,10, 5602-5610.
14. L. Ma, Y. L. Lv, B. W. Miao, G. R. Zhu, W. L. Liu, S. P. Guo, R. L. Tang, *Inorg. Chem. Front*. **2025**, DOI: 10.1039/d5qi00468c.
15. L. Q. Liu, H. L. Huang, M. L. Ding, Y. G. Shen, *Inorg. Chem*. **2024**, 63, 13835–13839.
16. J. Gou, Y. L. Zhu, X. Su, C. Yang, Y. J. Wang, Q. W. Zhu, Y. Xiong, Q. Wu, *Inorg. Chem. Front.* **2025**, 12, 3595-3601.
17. J. Chen, K. Z. Du, *Inorg. Chem*. **2022**, 61, 44, 17893–17901.
18. Q. Huang, C. L. Hu, B. P. Yang, Z. Fang, Y. Lin, J. Chen, B. X. Li, J. G. Mao, *Chem. Sci*., **2021**,12, 9333-9338.
19. Y. G. Shen, Y. W. Zhou, X. L. Xue, H. L. Yu, S. G. Zhao, J. H. Luo,  *Inorg. Chem. Front.* **2022**, 9, 5226-5230.
20. J. Z. Zhou, L. N. Wang, Y. Chu, H. S. Wang, S. L. Pan, J. J. Li, *Adv. Opt. Mater.* **2023**, 11, 2300736.
21. P. F. Li, C. L. Hu, J. G. Mao, F. Kong, *Chem. Sci.*, **2024**, 15, 7104-7110.
22. M. Yan, C. L. Hu, R. L. Tang, W. D. Yao, W. L. Liu, S. P. Guo, *Chem. Sci*., **2024**, 15, 8500–8505.
23. J. Chen, C. L. Hu, F. F. Mao, J. H. Feng, J. G. Mao, *Angew. Chem. Int. Ed*. **2019**, DOI: 10.1002/anie.201813968.
24. M. Yan, R. L. Tang, W. D. Yao, W. L. Liu, S. P. Guo, *Chem. Sci*., **2024**, 15, 2883–2888.
25. M. B. Xu, J. J. Li, H. Y. Wu, N. Ma, N. Yu, M. F. Zhuo, J. Chen, K. Z. Du, *Dalton Trans*., **2024**, 53, 10536–10543.
26. Y. H. Wang, F. Y. Li, D. X. Jiao, Q. Wei, L. Wei, G. Y. Yang, *Inorg. Chem*., **2023**, 62, 43, 17691–17696.
27. R. L. Tang, X. Lian, W. D. Yao, W. L. Liu, S. P. Guo, *Dalton Trans.*, **2021**, 50, 16562-16567.
28. M. J. Dodge, *Applied Optics*. **1984**, 23, 1980–1985.
29. S. H. Liu, L. L. Zhang, K. Li, X. T. Ma, X. F. Long, Y. Yang, *Dalton Trans*., **2025**, 54, 7214–7220.
30. W. Y. Zhang, Q. Jing, Y. Fang, *Z. Anorg. Allg. Chem.*, **2017**, 643, 1739–1743.
